# Microfluidic deposition for resolving single-molecule protein architecture and heterogeneity

**DOI:** 10.1038/s41467-018-06345-4

**Published:** 2018-09-24

**Authors:** Francesco Simone Ruggeri, Jerome Charmet, Tadas Kartanas, Quentin Peter, Sean Chia, Johnny Habchi, Christopher M. Dobson, Michele Vendruscolo, Tuomas P. J. Knowles

**Affiliations:** 1 Department of Chemistry, University of Cambridge, Lensfield Road, Cambridge, CB2 1EW UK; 2 WMG, University of Warwick, Coventry, CV4 7AL UK; 3 Cavendish Laboratory, University of Cambridge, Cambridge, CB3 0HE UK

## Abstract

Scanning probe microscopy provides a unique window into the morphology, mechanics, and structure of proteins and their complexes on the nanoscale. Such measurements require, however, deposition of samples onto substrates. This process can affect conformations and assembly states of the molecular species under investigation and can bias the molecular populations observed in heterogeneous samples through differential adsorption. Here, we show that these limitations can be overcome with a single-step microfluidic spray deposition platform. This method transfers biological solutions to substrates as microdroplets with subpicoliter volume, drying in milliseconds, a timescale that is shorter than typical diffusion times of proteins on liquid–solid interfaces, thus avoiding surface mass transport and change to the assembly state. Finally, the single-step deposition ensures the attachment of the full molecular content of the sample to the substrate, allowing quantitative measurements of different molecular populations within heterogeneous systems, including protein aggregates.

## Abstract (web summary)

Manual sample deposition on a substrate can introduce artifacts in quantitative AFM measurements. Here the authors present a microfluidic spray device for reliable deposition of subpicoliter droplets which dry out in milliseconds after landing on the surface, thereby avoiding protein self-assembly.

## Introduction

Atomic force microscopy (AFM) has emerged in the last decades as a powerful and versatile single-molecule analysis technique in biology, because it offers the opportunity of acquiring 3D morphological maps of specimens with subnanometer resolution, in both air and in liquid environment, helping to prevent denaturation^1–4^, unlike conventional scanning electron microscopy, which needs to be performed under condition of high-vacuum. Moreover, general AFM approaches have been extended to allow not only imaging of biomolecular samples, but also probing their mechanical properties, as well as the acquisition of optical spectra, offering unparalleled insights into the physical and structural properties of biological systems on the nanometer scale. This capability has been widely used in the fields of nucleic acid, protein and polymer science^2,4,5^. As a single-molecule technique, AFM is particularly valuable for investigations of heterogeneous biological systems, and it has in particular been successfully applied to unravel key aspects of the processes of protein aggregation and amyloid formation. Such processes are intimately related to the onset and progression of more than 50 human pathologies, including neurodegenerative disorders such as Parkinson’s and Alzheimer’s diseases^6,7^.

During the initial stages of amyloid formation, monomeric proteins misfold and form oligomeric species, which ultimately convert into mature fibrillar aggregates. An AFM morphology map provides extremely valuable information on the shape, size and heterogeneity of a solution of amyloid protein aggregates on the subnanometer length scale^7^. The technique offers the possibility to characterize with high statistical significance the distributions of the morphological properties of these aggregates, such as their height, diameter, periodicity, and flexibility. Such information is, for example, used for comparing the aggregation process of mutated forms of a protein, or to acquire information regarding the structural organization of mature fibrils^8–12^. The possibility of analyzing morphological properties at different time points provides information to clarify the mechanism of protein misfolding, the pathways of aggregation and the hierarchical polymorphic process of self-assembly^7,13^.

In a series of major advances, a range of AFM-based methodologies have recently been developed to correlate the morphology of protein aggregates with their fundamental biophysical properties, such as their chemical and secondary and quaternary structural organization^14^. These methods include quantitative nanomechanical mapping and analysis, tip enhanced Raman spectroscopy and infrared nanospectroscopy (AFM-IR)^7,15–20^. In these information-rich approaches the high-throughput investigation of heterogeneous protein solutions, however, commonly requires the analysis of the biophysical properties of samples in air environment^3,21^, indeed the application of these recently introduced advanced AFM modes is technically highly challenging and often not feasible in liquid environment^19,22–24^.

In recent years, despite such major advances in the development of new AFM modes and their applications, the science of sample preparation for AFM measurements has not changed significantly and limitations remain in the accuracy and reproducibility of this key step in the analysis. Previous studies have attempted to deposit biological samples by airbrush spraying, but these approaches were not capable of eliminating self-organization and artificial self-assembly of the molecules on the surface during drying^25,26^. A possible solution to this problem was proposed in the form of the use of artificial additives, such as glycerol, which reduced molecules mobility on the surface and reduced artificial self-organization;^27^ however, such additives result in non-native conditions for biomolecular studies. Furthermore, other studies have tried to deposit biological samples by spin coating, but this method has also been demonstrated to cause artificial self-assembly during drying as a function of the spin speed^28^. As such, currently, the process of biological sample deposition is still widely based on hands deposition and highly dependent on the manual skills of the operator^1,21,23,29,30^. A generic sample preparation process conceptually consists of three key steps: deposition of the sample onto a solid surface; buffer or water rinsing to detach weakly attached molecules, for measurements in liquid environment; and drying the sample for measurements in air^5,21,23,29^. These steps together take an amount of time between a few seconds and several minutes; such long time scales may lead to several artifacts that can often frustrates quantitative AFM studies. In particular, the accurate control of the quantity of biomolecules in the sample deposited on a surface is very challenging. In addition, during the time of physio-adsorption, the biomolecules can orient, align and self-assemble following the crystalline order of the surface^31–34^. Moreover, routine AFM measurements and analysis are limited to the use of only one substrate with a fixed charge state. Finally, the rinsing and/or the drying step do not guarantee that all the biomolecules originally in solution become attached on the surface at the end of the preparation; electrostatic repulsion and competitive binding between different species, for example, can reduce the fraction of adsorbed molecule on the surface^21^. These deposition artifacts are a primary cause of misinterpretation of the content and biophysical properties of heterogeneous biological systems, including in particular amyloidogenic proteins.

In the present work, we aim to address some of the key limitations of previous methods and to solve the long-standing problem of samples deposition on surface for microscopy and single-molecule studies by developing a microfluidic deposition approach. We demonstrate that a microfluidic 3D spray nozzle can be used as a reproducible, artifact-free, high-throughput and fully automated single-step method for depositing biological samples on solid substrates for single-molecule investigation by AFM in air. The device is able to spray onto the surface protein droplets of subpicoliter volumes and drying in times as short as fractions of milliseconds. Importantly, the timescale of droplet drying is comparable or much shorter than the theoretically and experimentally predicted time of the lateral diffusion of a monomeric protein or aggregate on a liquid–solid interface^34–37^. Thus, since proteins are unable to move freely, they cannot self-organize and self-assembly on the surface. In order to demonstrate the advantages of this method of sample preparation with respect to conventional manual deposition, we have used as a model system the deposition of a solution of monomeric and aggregated proteins onto atomically flat mica and highly ordered pyrolytic graphite (HOPG) surfaces. In particular, we have deposited α-synuclein, Aβ40 and Aβ42 peptides, whose aggregation is related to Parkinson’s and Alzheimer’s diseases.

## Results

### Surface interactions can bias manual sample deposition

**Fig. 1:**
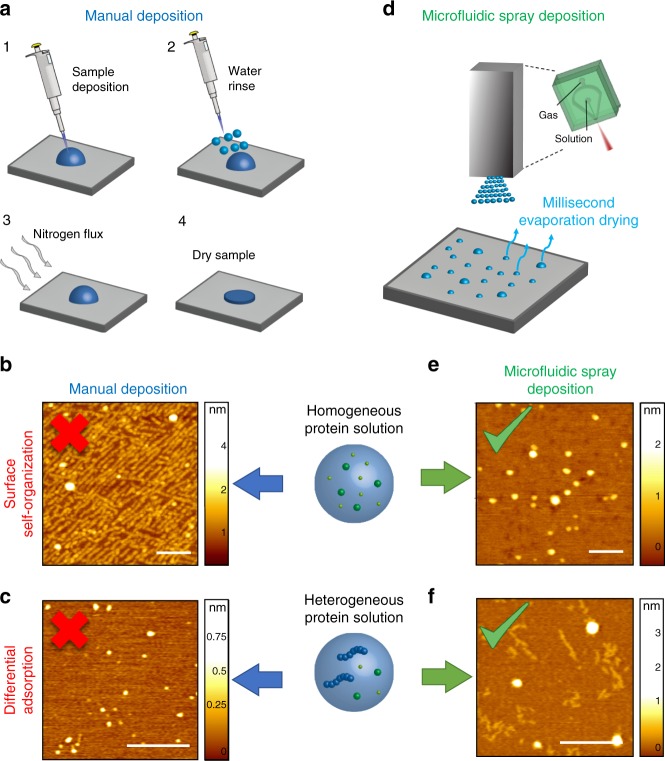
Manual vs. single-step deposition by microfluidic spray device. AFM sample preparation by (**a**) conventional manual deposition. This method may cause the (**b**) self-organization of monomeric α-synuclein along the crystallographic lattice of the mica substrate (scale bar 100 nm). **c** Furthermore, manual deposition typically enables only a partial depiction of a heterogeneous Aβ42 aggregated solution on a mica surface because of differential adsorption (scale bar 100 nm). While, (d) single-step microfluidic spray deposition (**e**) conserves the molecular architecture and assembly state of proteins in solution, and (**f**) enables to unravel the full content of the multiple protein species in solution (scale bars 200 nm)

The conventional manual deposition of samples on solid substrates for AFM imaging involves several steps, which are highly user dependent and require an operational time ranging from tens of seconds to minutes (Supplementary Note 1 and Supplementary Figure 1)^21,30^. In the case of preparation of a biological sample for measurements in ambient environment, as shown in Fig. 1a and Supplementary Figure 1, the process can be summarized in the steps of: (1) deposition of a droplet of solution with microliter scale volume on the substrate, (2) water rinsing, and (3) drying by means of a gas flux or an aspiration system^23,29^.

Commonly used surfaces are negatively charged mica, positively charged glass or gold substrates, and HOPG hydrophobic substrates^38–40^. Surface functionalization by means of a silane or divalent ions can be also exploited^8,17^. The manual preparation, because of the required long deposition and preparation times, can cause the self-assembly and reorganization of the sample on the surface (Supplementary Figs. 1–6). As example, in Fig. 1b, we show the self-organization of α-synuclein monomers along the crystallographic directions of the surface of a mica lattice. The self-assembly on the surface is clearly undesirable in the contest of clarifying the molecular architecture or the state of aggregation of biomolecules in the absence of surfaces. In addition, as result of rinsing and drying steps, when repulsive interactions exist between the substrate and the analyte, not all the biomolecules in solution could attach on the chosen substrate. In Fig. 1c, we show the manual deposition of a heterogeneous aggregated solution of Aβ42 protein, composed by oligomeric and protofibrillar aggregates. After manual deposition, only the oligomeric species are observable on the surface. Thus, the effect of selective absorption masks the effective heterogeneity of the deposited protein solution leading to a biased depiction of the composition and state of aggregation of the sample. Finally, the procedures of rinsing and drying together may result in a weakly reproducible control of the number and concentration of biomolecules deposited on the surface.

In order to standardize the preparation of AFM samples for measurement under ambient conditions and to overcome the limitations and artifacts of manual preparations, we have developed and used a microfluidics based 3D spray nozzle, which is represented in Fig. 1d. As demonstrated in the following paragraphs, the microfluidic spray device relies on the generation of subpicoliter droplets that evaporate: on a timescale comparable or shorter than the typical diffusion time of protein at liquid–solid interface, avoiding proteins self-organization and assembly on the surface; and before another droplet falls on the same area, thus preventing coalescence (Methods). As showed in Fig. 1e, d, the capabilities of the microfluidic spray deposition enable to avoid self-organization and differential adsorption as artifacts of deposition on the solid surface.

### Microfluidic spray device for AFM sample preparation

**Fig. 2:**
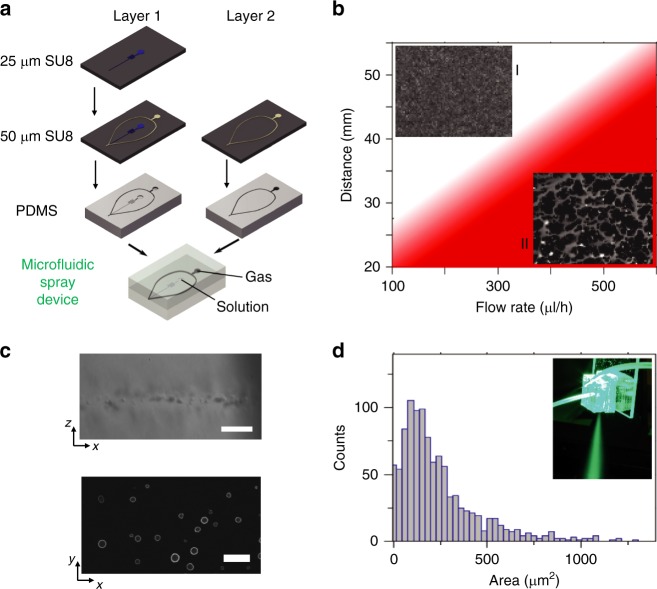
Single-step deposition by a microfluidic spray device. **a** Preparation of the microfluidic spray device. **b** Diagram representing the conditions, flow rate and distance from nozzle to substrate, resulting in well-resolved individual droplets (I) and streams of fluid due to coalescence (II), the insets in the diagram represent images of several layers of droplets for each condition. **c** Snapshots of real time videos showing deposition and drying of highly concentrated fluorescein droplets at the saturation point and in condition of absence of coalescence (scale bar: top, 50 μm; bottom 100 μm). **d** Histogram of the distribution of the size droplets imprints (*n* = 1032), the graph represents the distribution of the measured surface area of individual droplets, using a nitrogen pressure of 3.5 bar. The inset shows a photograph of the device in action

The microfluidic spray devices (Fig. 1d) were fabricated using soft-lithography techniques. The devices are composed of two complementary PDMS chips, which were produced using standard soft-lithography by replicating masters fabricated using a two-mask process based on fast wafer-scale light emitting diode (LED)-lithography patterning (Fig. 2a). The two PDMS chips are then activated with O_2_ plasma and put in contact with each other, with a drop of methanol in between them. The methanol drops were used to give a sufficient length of time before the bonding takes place to position the features precisely such that the two gas transporting channels are aligned (Fig. 2a)^41–43^. The solution carrying channels are 25 µm thick and 20 µm wide, while the final gas channels are 50 µm thick and 100 µm wide. The channels were connected through tubes to a syringe pump and a regulated pressurized N_2_ cylinder, respectively. The pneumatic spray atomization process relies on flow focusing of a narrow liquid stream flanked by pressurized gas to generate droplets as a result of Rayleigh–Taylor instabilities^44–46^. This mechanism of hydrodynamic focusing is known to induce low-viscous shear stresses in the liquid^44^. Thus, the method is well suited for the deposition of a range of fragile molecular species, including biomolecules and other complexes. The deposition of the samples was realized by placing the microfluidic spray device perpendicularly to the surface of deposition, at a fixed distance (2.5–5 cm) and spraying at a constant flow rate (100–300 μl h^−1^). The deposition time for the microfluidic spray device corresponded to the time of spraying on the substrate, which was precisely controlled by using a shutter mounted on a stepper motor controlled by an Arduino Uno to stop the spray. The volume of the solution sprayed on the surface ranged approximately between 0.02 and 1 μl for a time of deposition between 1 and 60 s.

First, a model solution of fluorescein protein in water was used as system to estimate the time of drying of the amyloidogenic proteins during the time of flight and after landing on the substrates (Methods). This model system was used to visualize by an ultrafast camera the landing of the droplets, visualize their imprints (Fig. 2c), evaluate the regime of noncoalescence of the droplets and estimate their volume from their area on the surface (Fig. 2d and Methods).

The deposition of the amyloidogenic protein samples was realized at a distance in average of 4 cm, and spraying at a constant flow rate of 100 μL h^−1^ (Fig. 2b), which corresponded to a regime of noncoalescence of the droplets (region I in Fig. 2c, Methods). We exploited the theory of evaporation of a droplet during the time of flight in air and on a surface to estimate the average time of drying. For a typical droplet of protein solution generated by the microfluidic spray device, we estimated an average time of drying on the surface of 2 ms, with an interquartile range between 0.7 and 4.3 ms (Supplementary Figure 2 and Supplementary Notes 1–2). An intrinsically disordered protein, represented as polypeptide chain of approximately 10 nm diameter, or a larger amyloidogenic aggregate, have a typical lateral diffusion constant on the solid–liquid interface of 0.1–0.3 μm^2^ s^−1^. In an interval of time of 0.7–4.3 ms, considering the 2D diffusion as an upper bound on displacement on the surface, the molecules could displace at maximum of a distance of approximately 40 ± 20 nm (interquartile range, Supplementary Figure 2 and Supplementary Note 3). The diffusion length is calculated considering that the protein are in contact with the surface for the full time of drying, thus it is an unfavorable case. Indeed, once a droplet has landed on the surface, first protein need to diffuse towards the interface and only later the 2D diffusion can occur (Supplementary Note 3). In conclusion, the time of drying of the subpicolitre droplets is always comparable to or smaller than the minimal reduced time of a diffusion step of a protein, the deposited monomeric and aggregated proteins are not able to move during the deposition for a significant distance and are rapidly fixed onto the surface (Methods, Supplementary Note 4 and Supplementary Figure 2)^36,47–49^.

### Microfluidic spray deposition of monomeric protein solutions

**Fig. 3:**
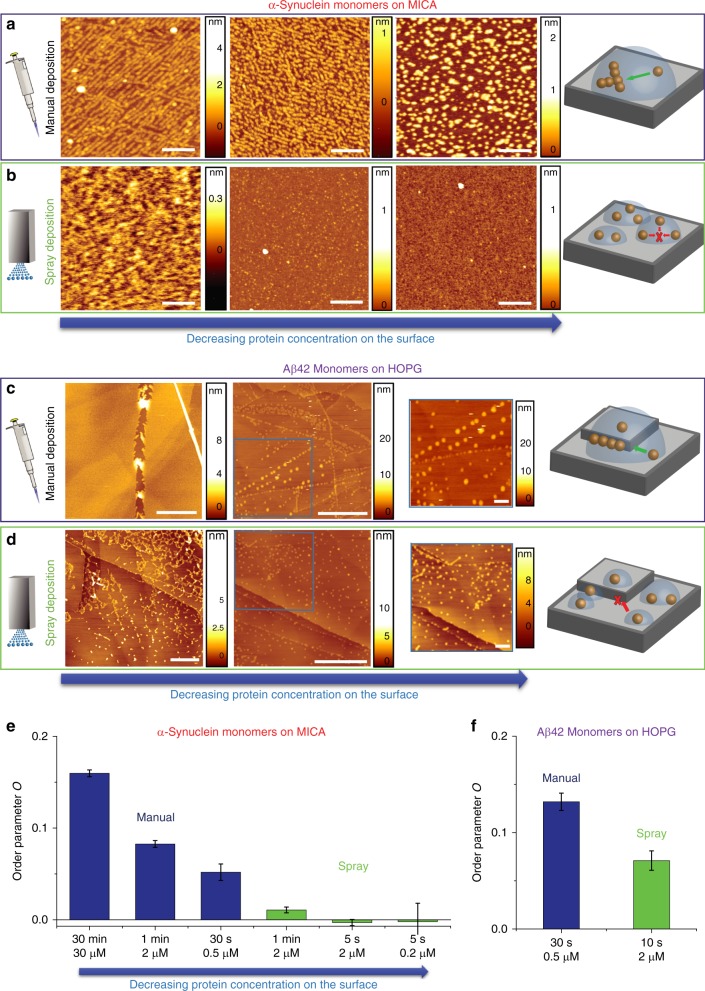
Rapid drying by the microfluidic spray technique avoids surface self-organization. Deposition of α-synuclein monomers on a mica surface by (**a**) manual preparation at decreasing protein concentration of deposition on the surface: 30 μM and 30 min, 2 μM and 1 min, 0.5 μM and 30 s (scale bars 100 nm); (**b**) microfluidic spray of a 2 μM solution for 1 min (scale bar 100 nm) and 5 s, 0.2 μM for 5 s (scale bars 100 nm). Deposition of Aβ42 monomers on a HOPG surface by (**c**) manual preparation at decreasing protein concentration on the surface: 2 μM and 1 min (scale bar 500 nm) and 0.5 μM and 30 s (scale bar 1 μm); (**d**) microfluidic spray of a 2 μM solution for times of 1 min (scale bar 500 nm) and 10 s (scale bar 1 μm). On the right side of each panel, a detail of the AFM maps (scale bar 100 nm) and illustrations of the allowed diffusional processes of individual monomers are presented. Quantification of the degree of ordering, through the order parameter O, in each AFM map for (**e**) α-synuclein and (**f**) Aβ42 (values are mean ± s.d.)

We sought to demonstrate that the spray device can advance the preparation of AFM samples for measurement in air while avoiding the artifacts of sample organization and self-assembly on the surface subsequent to deposition (Supplementary Figure 1a). To probe the capabilities of our device, we compared manual and microfluidic spray mediated sample preparation using as model systems monomeric α-synuclein and Αβ42 on mica and HOPG, respectively (Fig. 3). We chose to probe independently the advantages of our device with two commonly used substrates and proteins inherent in the context of aggregation related neurodegenerative disorders.

In Fig. 3a, we represent the manual deposition of α-synuclein on mica (Supplementary Figures 1, 3, and 4). For long deposition times and a highly concentrated solution (30 min and 30 μM), which are normally required to strongly attach the molecules before than rinsing and drying, the proteins are observed to self-organize and self-assemble, forming fibril-like aggregates, along the positive sites of the cleavage of mica. Although reducing the deposition time and concentration to 1 min and 5 μM reduced the effect, self-organization was evidently taking place. Only by performing the deposition at a sub-μM concentration (0.5 μM) and rapidly (30 s), in a time that is close to the limit of capabilities of a human operator and to the minimal one required to allow physio-adsorption, it was possible to avoid the self-assembly of the proteins, although, even in this case, interaction and co-localization was clearly still evident. In Fig. 3b, we show examples of the spray deposition of α-synuclein monomers using the microfluidic device developed in this work. The protein solution was sprayed at a constant speed of 100 μl h^−1^ at concentration of 0.2 μM and 2 μM. For relatively long deposition times (above 1 min for 2 μM), corresponding to high concentrations of protein on the surface, the monomers formed a continuous layer on the mica surface with no visible self-organization and self-assembly on the surface. Decreasing the spraying time to 5 s, thus reducing the amount of the deposited protein, we could observe individual monomeric species and their concentration on the surface could be controlled without any evidence of deposition artifacts. Similarly, in Fig. 3c, examples of the manual deposition of the Aβ42 peptide onto HOPG surface are illustrated. At high concentration and long deposition times (2 μM for more than 1 min), the monomeric protein formed a continuous layer, self-organizing on the surface showing several circular rings following the hexagonal geometry of HOPG (Supplementary Figure 5). Reducing the deposition time and concentration to 30 s and 0.5 μM, respectively, enabled visualization of individual monomeric and early oligomeric species. However, during the deposition time, the proteins were free to diffuse and self-organized along the steps of HOPG, which acted as free ledge and kink sites^31,50^. In contrast, in Fig. 3d, we show the spray deposition of a solution of Aβ42 at a concentration of 2 μM and at a speed of 100 μL h^−1^. For deposition times above 1 min, generating high protein concentration on the surface, the molecules were partially interacting. For short deposition times (e.g., 10 s) and thus at low concentration, the molecules were uniformly spread over the surface; indeed, there was no evidence for self-assembly and for organization along the steps of the different HOPG planes.

The comparison of the two experimental methods, as illustrated on the right side of Fig. 3, confirms that during the several seconds needed for manual deposition, the protein molecules can diffuse and interact among themselves and with the surface, therefore they self-organize and self-assemble (Fig. 3a, c). Crucially, the preparation of the samples using the microfluidic spray technique enables a very rapid drying of the sample droplets, thus avoiding diffusion over the surface and resulting in significantly reduction of self-organization and ordering on both negative mica and hydrophobic HOPG surfaces. To prove quantitatively that the molecules do not self-organize during the deposition, we developed an ad hoc analysis of the images that evaluates the artifacts of alignment and self-organization of monomeric α-synuclein and Aβ42 proteins over the surface of deposition by means of the order parameter *O* (Methods and Supplementary Figure 7). A higher value of *Ο* is correlated to a more ordered sample. In Fig. 3e, we present the results of the application of this method of analysis of the images, showing the degree of ordering corresponding to the AFM maps of α-synuclein as a function of protein concentration on the surface (Fig. 3a, b), and in Fig. 3f the degree of ordering of Aβ42 at low concentrations on the surface (Fig. 3c, d).

**Fig. 4:**
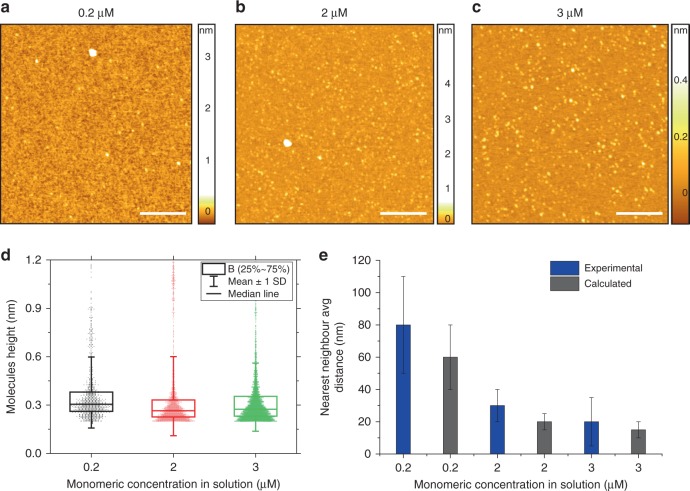
Microfluidic spray deposition avoids artificial self-assembly and enables accurate tuning of molecules concentration on the surface. AFM maps of the microfluidic spray deposition of a monomeric solution of α-synuclein at (**a**) 0.2 μM, (**b**) 2 μM, and (**c**) 3 μM for 5 s at 100 μl h^−1^ (scale bars 200 nm). **d** Box chart distributions of the cross-sectional height of the deposited biomolecules is independent from the concentration of deposition (*n* = 1003 0.2 μM, *n* = 2441 for 0.5 μM, *n* = 3685 for 3 μM; the horizontal line represents the median, the box the first to third interquartile, and the error is the s.d.). **e** Average molecular distance on the surface (average nearest neighbor) decreases as a function of the concentration of the deposited solution, in excellent agreement with theoretical calculations (values are mean ± s.d.)

In order to demonstrate that the drying process during the microfluidic spray of the droplets is not affecting the assembly state of the biomolecules in solution, inducing oligomeric or fibrillar aggregation, we sprayed a monomeric solution of α-synuclein varying protein concentration in solution over more than an order of magnitude (0.2 μM, 2 μM and 3 μM) (Fig. 4a–c). A statistical analysis of the cross-sectional dimensions of the deposited molecules showed the presence of a constant population of species with cross-sectional height of approximately 0.2–0.6 nm, independently from the concentration of the sample, as expected for a monomeric sample of α-synuclein (Fig. 4d and Supplementary Figure 8)^8,17,51^. These results demonstrated that the microfluidic spray deposition preserves molecular architecture and does not induce oligomeric aggregation during drying of the droplets, besides we could not observe any induced aggregation of fibrillar species on the surface.

Furthermore, in the AFM maps in Fig. 4a–c, it is possible to observe an increased number of proteins on the surface as a function of the increasing concentration. In order to quantify the capability of the microfluidic spray device to tune accurately the number of molecules deposited, we considered the average nearest neighbor distance in the AFM maps as an indicator of the surface occupancy (Fig. 4e). We observed that the distance between the deposited molecules decreased as a function of the concentration of the solution deposited. The measured intermolecular distance as a function of the concentration was in excellent agreement with a theoretical modeling of the process of deposition (Supplementary Note 4), thus demonstrating the capability of the spray to tune accurately the number and concentration of biomolecules on the surface and to avoid artifacts related to surface overcrowding (Supplementary Figure 1).

### Microfluidic spray deposition avoids differential adsorption

The study of heterogeneous biological samples by AFM is often severely hampered by the forces regulating the adsorption of proteins at liquid–solid interfaces. Indeed protein solutions, such as for example those from amyloidogenic systems, commonly contain several species possessing different charge states. During manual preparation of AFM samples, the rinsing and drying of the sample can prevent the adsorption on the surface of some of the molecular species in solution, because of electrostatic repulsive forces. As result of such interactions, selective adsorption results in only a partial representation of the actual sample composition (Fig. 1b and Supplementary Figure 1). We demonstrate below that the automated microfluidic spray deposition of samples relies on a single deposition event, eliminating the rinsing and drying steps and retrieving the complete heterogeneity of even complex protein samples.

**Fig. 5:**
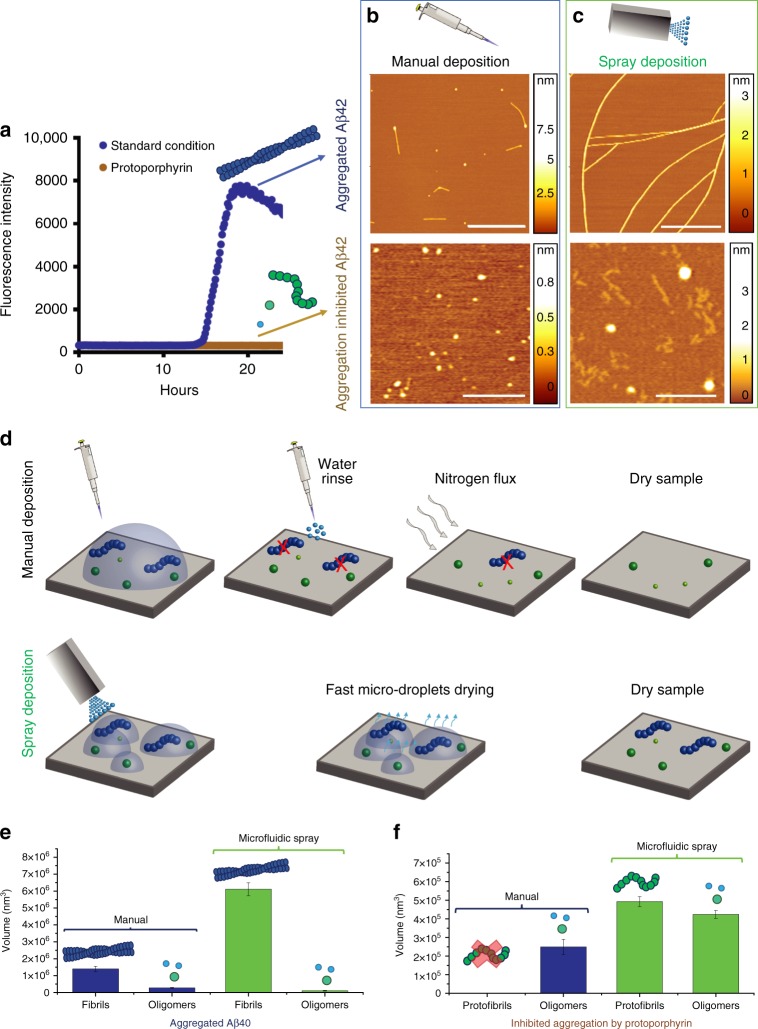
Spray deposition avoids selective sample adsorption. **a** Kinetic profiles of the aggregation reaction of 10 μM Aβ40 under standard conditions (blue curve) and in the presence of protoporphyrin (Proto), which inhibits its aggregation (brown curve). Deposition of the final products of the aggregation in the absence and presence of the inhibitor by (**b**) manual (scale bar: top image 1000 nm, bottom image 200 nm) and (**c**) automated spray deposition (scale bar: top image 1000 nm, bottom image 200 nm). **d** Illustration of the differences between the two preparative methods and the effects leading to selective adsorption. Quantification of the absolute mass (values are mean ± s.d.) of coexisting amyloid species at the end of the aggregation reaction by measuring the volume of each species in (**e**) standard and (**f**) inhibited conditions of aggregation

In order to demonstrate the advantages of spray above manual deposition, we studied Aβ40 in the amyloid aggregated state and in conditions where aggregation was inhibited by the small molecule protoporphyrin. In Fig. 5a, we show the aggregation kinetics of Aβ40 monitored by means of the increase in fluorescence of the amyloid specific dye Thioflavin T (ThT)^6^. Under standard conditions (Methods) the protein shows a typical nucleation growth curve with a sigmoidal shape of the curve, while in the presence of protoporphyrin the aggregation process is inhibited, as indicated by the lack of ThT signal. An SDS–PAGE analysis further reveals that mature Aβ40 fibrils, which are usually collected in the pellet fraction after centrifugation, are no longer present when the aggregation is performed in the presence of protoporphyrin (Supplementary Figure 9). In Fig. 5b, we show results from the manual deposition of aliquots from the sample at the two final points of the aggregation process. In the case of Aβ40 alone, we can observe several small fibrils with a typical length of 1 μm (Supplementary Figure 10). By contrast, in the presence of the inhibitor, we can observe only oligomeric species, a result that might be thought to reflect reasonably the bulk measurements. However, as Fig. 5c reveals, the images of samples from the same solution provided by the microfluidic spray device show a striking misinterpretation of the data. The sample of Aβ40 now shows long branched fibrils with lengths of the order of several micrometers, and the sample aggregating in the presence of the inhibitor shows the simultaneous presence in solution of both oligomeric and protofibrillar species, which were not detected by the manual preparation. The protofilaments showed a typical cross-sectional length in the order of hundreds of nanometers (Fig. 5c bottom). We verified the heterogeneity of the sample independently by dynamic light scattering (DLS). This bulk technique showed the presence of aggregated species with hydrodynamic radii of 50–500 nm (Supplementary Figure 9) in excellent agreement with the AFM measurements obtained by microfluidic spray deposition.

The rationalization of the improved capabilities of the automated spray deposition technique, relative to the manual equivalent, is shown schematically in Fig. 5d and quantified in Fig. 5e, f, where the relative abundance of the oligomeric, protofibrillar and fibrillar species is measured as the total volume occupied for a given unit of surface area. The manual processes of rinsing and drying of the surface remove all the molecules not interacting strongly with the surface (Fig. 5d). In both cases of normal and inhibited aggregation, the measurement of the volume of the aggregated species on the surface reveals that the manual preparation reduces the total number of aggregates physio-adsorbed on the surface, when compared to microfluidic preparation (Fig. 5e, f). In addition, the manual deposition does not allow the presence of the protofibrillar population to be revealed in the case of inhibited aggregation by protoporphyrin.

Furthermore, we aimed at comparing the microfluidic spray deposition with spin coating deposition. We performed the deposition of fibrillar species by spin coating at two different speeds, 1000 and 3000 rpm (Supplementary Figure 11). In agreement with previous studies attempting spin coating deposition of protein samples, we observed the artificial formation of regular globular structures with 1–4 nm diameter on the surface, which decrease in abundancy and size with increasing spin speed^28^. Indeed, spin coating requires the deposition of an excess of solution to coat a surface for at least several seconds, thus still allowing diffusion on the surface and self-assembly that depend on the spin speed and the duration of the process. Critically, the artificial globular aggregates also form on the fibrillar species preventing the possibility to characterize their morphology. When compared to microfluidic spray deposition, where droplets drying in milliseconds without excess solution (Supplementary Note 6 and Supplementary Figure 12), the presence of artificially self-assembled globular aggregates, which vary morphology and abundancy as a function of the spin speed, excludes the possibility for samples prepared by spin coating to evaluate the state of aggregation, heterogeneity and the morphology of the protein species in solution. Furthermore, solution loss during the spinning does not allow characterizing the sample heterogeneity and quantification of the amount of sample deposited. By contrast, the microfluidic spray deposition technique is capable of depositing the full content of the solution, avoiding any artificial self-assembly and self-organization on the protein on surface, resulting in all molecular species being deposited.

Finally, the characteristics of the microfluidic spray deposition of preserving molecular architecture and heterogeneity, coupled with the possibility of controlling accurately the volume and number of deposited proteins on the surface, offers the possibility to tune reproducibly their concentration on the surface, which is also of paramount importance to minimize the use of costly protein, such as samples extracted from human patients.

## Discussion

Preparation of biological and protein samples for AFM investigations under ambient conditions is of fundamental importance for the robust, artifact-free and high-throughput measurements of their morphological and intrinsic biophysical properties, such as internal structural organization and nanomechanical properties. The importance of reliable sample preparation methods is further highlighted by recently introduced innovative AFM-based methods that bring together optical or mechanical spectroscopy with scanning probe measurements and often require operation in air^7^.

In this work, we have demonstrated that a microfluidic spray device can be used for the automatic, reproducible and single-step deposition of biological samples for AFM measurements in air. The device, denoted microfluidic spray device, relies on flow focusing and Rayleigh–Taylor instabilities to produce with low shear forces droplets of subpicolitre volume, which can be sprayed onto a surface at constant speed by means of a pressurized control system. The droplets dry directly on the surface in times as small as submilliseconds. The time of drying is faster than the time before another droplet falls on the same area, thus preventing droplets coalescence and than the diffusion mobility times of monomeric and aggregated proteins on a liquid–solid interface^36,37,48^. This fast drying time, on average a million times faster than the time needed in previous manual deposition methods, including airbrush spray and spin coating deposition^25,28^, reduces dramatically the self-organization and avoids artificial self-assembly of the sample on the surface. In addition, the direct drying of protein samples on the surface enables the investigation of heterogeneous systems free of artifacts, and eliminating the problem of surface-sample selective adsorption. Finally, when compared to previous hand-operated methods of sample preparation, the capabilities of the microfluidic spray device enable accurate control of the amount of protein deposited onto a surface, which can be as little as 0.01 μl, minimizing the usage of costly and valuable proteins, and the full deposition of the content of a solution.

This method paves the way for automated, artifact-free and high-throughput preparation of samples, not only for AFM measurements, but in general for scanning probe microscopy and imaging techniques and thus generates the possibility of acquiring fully single-molecule quantitative data on heterogeneous molecular samples. Moreover, in addition to applications for atomic force microscopy studies, our results provide the basis for establishing a reproducible and quantitative single- step deposition method for conventional electron microscopy studies, which are carried out in vacuum and thus by their very nature rely on sample deposition and drying processes.

## Methods

### AFM measurements

Atomic force microscopy was performed on bare mica and HOPG substrates. AFM maps were acquired by means of a Multimode VIII (Bruker), a NX10 (Park systems) and a nanowizard2 (JPK) system operating in tapping mode and equipped with a silicon tip (μmasch, 2 Nm^−1^) with a nominal radius of 10 nm. Image flattening and single aggregate statistical analysis, such as nearest neighbor and cross-sectional dimension analysis, were performed by SPIP (Image Metrology) software.

### Device preparation and materials

The Polydimethylsiloxane (PDMS) molds are produced in two steps using SU-8 photolithography using UV LED. The two PDMS chips are then activated with O_2_ plasma and put in contact with each other, with a drop of methanol in between. (Diener etcher, Femto, 40% power, 30 s). The methanol is used to give enough time before the bonding takes place to position the features precisely such that the two gas transporting channels are aligned.

### Preparation of monomeric Aβ42 and α-synuclein solutions

Solutions of Aβ42 were prepared by dissolving the lyophilized protein in 6 M GuHCl. Monomeric forms were purified from the presence of potential oligomeric species and salts using a Superdex 75 10/300 GL column (GE Healthcare) at a flow rate of 0.5 mL min^−1^, and were eluted in 20 mM sodium phosphate buffer, pH 8 supplemented with 200 μM EDTA and 0.02% NaN_3_. The center of the peak was collected and the Aβ42 concentration was determined from the absorbance of the integrated peak area using *ε*_280_ *v* = 1490 l mol^−1^ cm^−1^. Recombinant α-synuclein was synthetized in *E. coli* and then purified by previously accepted protocols^52^. The monomeric protein samples were filtered (>95%) in a solution in a 50 mM TRIS-buffer, NaCl 150 mM and 7.5 pH and incubated at 37 °C to form prefibrillar and fibrillar aggregates.

### Preparation aggregated Aβ40 samples

Chemicals, including protoporphyrin IX, were obtained from Sigma-Aldrich and were of the highest purity available. Recombinant Aβ40 peptide was expressed and purified as in the case of Aβ42, as explained above^53^. Protoporphyrin IX was suspended in 100% DMSO at 5 mM, then diluted in the Aβ40 solution to reach a final DMSO concentration that did not exceed 1%. The samples (10 μM Aβ40 in the absence and presence of 5 μM protoporphyrin IX) were prepared in low-binding Eppendorf tubes on ice, using careful pipetting to avoid the introduction of air bubbles. They were then pipetted into multiple wells of a 96-well half-area, low-binding, clear bottom and polyethylene glycol coating plate reader (Corning 3881), 80 μL per well. The samples were also supplemented with 20 μM ThT from a 2 mM stock solution to follow the aggregation reaction to completion. The samples were then collected back into low-binding Eppendorf tubes. For the sodium dodecyl sulfate–polyacrylamide gel electrophoresis (SDS–PAGE) analysis, samples were centrifuged at 9400×*g* for 15 min at 25 °C. Pellets and supernatants were then separated, and the pellets were resuspended in the same volume of buffer as that of the supernatant. These fractions were then analyzed using SDS–PAGE.

### Dynamic light scattering (DLS)

DLS experiments were performed with a Zetasizer Nano-S (Malvern) at 25 °C using preformed 10 μM Aβ40 fibrils in 20 mM phosphate buffer at pH 8 either in the absence or presence of 5 μM protoporphyrin IX. All calculations were performed using the software provided by the manufacturer.

### Spraying regime and droplet evaporation time

A fluorescein solution was used as model system to gain insights into the characteristics of the spray nozzle and in particular to measure the volume, time of drying of the droplets and to find the conditions to avoid coalescence.

The study of the imprints of individual droplets enabled the estimation of the droplet volume and their distribution on the surface of a glass slide using an epifluorescence microscope. A high concentration of fluorescein, at its limit of solubility, was exploited to visualize the border of the area of the droplets on the surface through the coffe-ering effect (Fig. 2c, d). In order to obtain images with individual droplets only, the spray was applied for 200 ms. More than 20 images were taken for different spraying conditions and the software *ImageJ* was used to calculate the surface area left by individual droplets on the substrate. In the spraying regime, defined by pressures above 2 bar and the noncoalescence condition (corresponding to area I in Fig. 2b), the mean size of the droplets imprints does not vary significantly with the flow rate or the gas pressure and had a typical surface between 50 and 250 μm^2^. The shape of the droplets on the substrate depends on their equilibrium contact angle. For small droplets, the drop shape can be taken as a portion of a sphere if the gravitational forces can be neglected. This is the case if the Bond number, given by *B*_*o*_=*ρgL*^2^γ^−1^, where *g* is the acceleration of gravity, is sufficiently small (*B*_*o*_ ⪡ 1), a condition fulfilled for the droplets generated using the spray nozzle presented in this study. A contact angle of 15.3° (SD = 2.6°) was measured for the model fluorescein solution (1 µL droplets) on the glass substrates used. In general, for droplets with a contact angle smaller than 90°, it is expected that the contact line gets pinned during evaporation^54^. This situation was verified experimentally using contact angle measurements. Therefore, the imprints observed on the images represent the base of the droplets upon landing on the substrate, making it possible to calculate the volume of each droplet using the equilibrium contact angle. Starting from the measurement of the surface area of the imprints (Fig. 2d), in the case of fluorescein sprayed 2 cm away from the surface, we could estimate that the volume of the droplets on the surface had a median value of 100 fL and an interquartile range between 40 and 210 fL, with a volume of 1 fL for the smallest droplets observed. For fluorescein droplets with a volume of approximately 100 fL and sprayed from a distance of approximately 2 cm, we measured by high-speed camera videos an average value of the time for complete drying on the surface of about 5 ms, in agreement with previous studies for droplets of modest height to radius ratio^55^. On the base of previous results on the time of drying of sessile droplets on a surface, we can generally assume that the time of drying of the fluorescein droplets is similar to the one of the sprayed amyloidogenic monomeric and aggregated proteins in Figs. 3 and 4^55^.

In particular, in the case of the amyloidogenic proteins, in order to further reduce the concentration of the droplets on the surface for single-molecule studies, were sprayed from a longer distance, of approximately 4 cm, which was in average 2 cm longer than in the case of fluorescein for measuring the drying time. To correctly evaluate a relationship between the radius of a droplet and its evaporation time on the surface, we had to consider both the evaporation during the time of travel toward the surface and the evaporation of the sessile droplet on the surface (Supplementary Figure 2). Indeed, because of the longer distance from the surface, the droplets of protein solution landed on the surface have smaller volume and quicker evaporation time than fluorescin droplets. In our conditions, the classical Maxwell’s theory of evaporation of a droplet in air establishes that the evaporation rate of droplets during the time of flight is in the order of 1 × 10^−3^ms^−1^, which is circa 10 fL ms^−1^^56^. Furthermore, by means of a high-speed camera, we could measure an approximate velocity of our droplets in the order of 10–30 m s^−1^. The time of travel and the time of evaporation on surface have similar order of magnitude and need to be taken in account to evaluate the final dimensions and time of drying of the droplet on the surface (Supplementary Note 1–2). We concluded that the final volume of droplets of amyloidogenic proteins on the surface had a median volume of 25 fL, with interquartile range between 5 and 80 fL, and drying in a time ranging between 0.7 and 4.6 ms (Supplementary Note 1–2 and Supplementary Figure 2)^55^. Temperature and humidity are important factors affecting the droplet evaporation rate. We have showed that a 2 °C and a 10% relative humidity variation (under standard air-conditioned laboratory conditions) can cause a change in the droplet drying time on the surface under 1 ms. This time variation is not significant because we have a poly-disperse droplet distribution with a range of drying times larger than the variation introduced by temperature and humidity, which thus do not play an important role in defining the time of drying during the sample deposition on surface in our conditions (Supplementary Note 2).

Finally, we demonstrated the regime of noncoalescence of the droplets (Supplementary Note 5). If the spray is close to the substrate and the flow rate too high, the deposited droplets do not have time to evaporate before incoming droplets reach the same area. In this case, the droplets coalesce to form continuous liquid streams. By increasing the distance between the nozzle and the substrate, it is possible to apply high flow rates without observing the adverse effect of coalescence. The reason no coalescence is observed in this case is due to the fact that the density of droplets landing on the surface decreases with the distance of the nozzle, thus minimizing the coalescence probability. The typical spraying cone was of 18° (SD = 2.4°) was measured for an applied pressure of 2.5 bars and a flow rate of 300 µl h^−1^, and did not change significantly under other conditions. Figure 2b shows a diagram representing the flow rate and distance conditions to avoid coalescence (area I). In the case of fluorescein, for droplets below 250 fL (representing an unfavorable case) and drying time in about 10–15 ms, considering a flow rate of 300 µl h^−1^, we had approximately 7000 droplets that cover less than 0.3% of the total surface reached by the spray. Therefore, the probability of coalescence is literally null, as confirmed by our observations and in agreement with theoretical calculations (Supplementary Note 5).

### Measure of degree of assembly and ordering on the surface

In order to characterize and evaluate quantitatively the possible degree of ordering and alignment of the proteins on the surface, because of mass transport phenomena during drying, we developed an ad hoc customized quantitative image analysis of the AFM 3D morphology maps in Fig. 3. The goal is to detect molecules (maxima in the *Z*-direction) that are organized along a line. First, the maxima are localized in the image by ImageJ software. Then, an angle distribution is extracted from these positions. Finally, the ordering is estimated by comparing the angle distribution of the image with the angle distribution of a set of positions distributed randomly on a surface.

The molecules are localized in the AFM maps using the Maxima Finder algorithm of the ImageJ software (Supplementary Figure 7)^57,58^. The images are filtered with a median filter to reduce noise before to localize the maxima. The software uses a maximum filter to find the local maxima and a watershed algorithm to control if another maxima could be reached within a given noise tolerance. A threshold on the pixel value is used to avoid getting false positives. The maxima and the positions satisfying these conditions are saved in a.csv file. An angle distribution is computed from the pairs of nearest neighbors, which are found by using a KDTree algorithm^59^. For each maxima position $\overrightarrow {{\boldsymbol{x}}_{\boldsymbol{i}}}$, the nearest neighbor $\overrightarrow {{\boldsymbol{x}}_{{\boldsymbol{i}},1}}$ is found and then the vector $\overrightarrow {{\boldsymbol{p}}_{\boldsymbol{i}}} = \overrightarrow {{\boldsymbol{x}}_{\boldsymbol{i}}} - \overrightarrow {{\boldsymbol{x}}_{{\boldsymbol{i}},1}}$ is computed. If the pair was already found with $\overrightarrow {{\boldsymbol{x}}_{{\boldsymbol{i}},1}}$ as a center, it is not double counted. If $\left\| {\overrightarrow {{\boldsymbol{p}}_{\boldsymbol{i}}} } \right\|$ is larger than the distance of $\overrightarrow {{\boldsymbol{x}}_{\boldsymbol{i}}}$, to one border of the image, the pair is discarded to avoid border effects on ordering. The angle corresponding to each pair, which is the angle between $\overrightarrow {{\boldsymbol{p}}_{\boldsymbol{i}}}$ and the ${\hat{\boldsymbol x}}$ axis, is:

$$\theta _i = \arctan \left( {\frac{{p_{i,y}}}{{p_{i,x}}}} \right)$$

and it has a value ranging between [−π/2;π/2]. Then, all the angles *θ*_*i*_ are organized in a histogram, which we called the angle distribution (Supplementary Figure 7). The number of bins is limited by the total number of maxima in an image. Indeed, some images have as few as 100 visible maxima. In order for each bin to have at least several tenths of counts, the analysis is performed with *N*_bin_ = 8 bins. Each bin is centered on (*nπ*/8), which is a multiple of $\pi /8$ with *n* between −4 and +4. Two of the bins, centered at −π/2 and π/2, being only half filled, are combined. Indeed, only the orientation, and not the direction, is relevant. The bins are, therefore, aligned with the principal directions of the pixel grid, which minimizes the artifact caused by the discrete nature of the pixels positions in the AFM map.

If the points are randomly distributed, the angle distribution histogram is expected to be flat as all the angles are equally probable. By contrast, if there is ordering in the image, some angles between nearest neighbors would be more probable and some of the bins in the angle distribution will be over-filled, while some others will be under-filled. Thus, the presence of ordering in the image would cause a deviation of the angle distribution from the flat behavior of the angle distribution of a set of points randomly distributed on the surface.

A statistical analysis is carried out on the histogram of the angle distribution of the molecules in each image to separate a random variation from a deviation because of ordering and alignment on the surface. The mean and variance associated with each bin of the histogram are given by the binomial distribution: *μ*_bin_ = *Np* and $\sigma _{\mathrm{bin}}^2 = Np\left( {1 - p} \right)$, respectively. *N* is the total number of maxima and $p = 1/8$ is the probability for an angle to be in each bin. For a histogram with a count $C_i$ in each bin, the total sample variance $\sigma _{\mathrm{Sample}}^2$ of the angle distribution is estimated as:

$$\sigma _{{\mathrm{Sample}}}^2 = \frac{1}{{N_{{\mathrm{bin}}}}}\mathop {\sum }\limits_i^{N_{\mathrm{bin}}} \left( {C_i - \mu _{{\mathrm{bin}}}} \right)^2$$

If the molecules are ordered on the surface, $\sigma _{{\mathrm{Sample}}}^2$ might be significantly larger than the variance of the angle distribution expected from the distribution of the same number of angles $\sigma _{{\mathrm{bin}}}^2$, between pairs of molecules randomly distributed on a surface. This excess of variance is used as a parameter to quantify the degree of ordering. Since the ordering is a signal and not a noise, the variance will scale with $\mu _{{\mathrm{bin}}}^2$ and needs to be normalized. Therefore, we define the ordering parameter *O* (Supplementary Figure 7) as the normalized difference of the sample variance of the angle distribution of a real image, $\sigma _{{\mathrm{Sample}}}^2$, against the value of the variance of the angle distribution expected from a random distribution $\sigma _{{\mathrm{bin}}}^2$:

$$O \equiv \frac{{\sigma _{{\mathrm{Sample}}}^2 - \sigma _{{\mathrm{bin}}}^2}}{{\mu _{{\mathrm{bin}}}^2}}$$

The variance of the angle distribution of a sample can be smaller than the theoretical variance as only a finite number of points are considered. Therefore, if the sample has a very small degree of ordering (Fig. 3), the ordering parameter can be negative. Any negative values from this algorithm are expected to be within the standard deviation of the ordering parameter.

Using the central limit theorem, we can estimate the variance of the variance of the angle distribution of the sample:

$${\mathrm{Var}}[\sigma _{{\mathrm{Sample}}}^2] = \frac{{2\sigma _{{\mathrm{bin}}}^4}}{{N_{{\mathrm{bin}}}}}$$

Therefore, the standard deviation of the ordering parameter $O$ is:

$$\sigma _O = \frac{{\sigma _{{\mathrm{bin}}}^2}}{{\mu _{{\mathrm{bin}}}^2}}\sqrt {\frac{2}{{N_{{\mathrm{bin}}}}}}$$

For images with a low number of maxima, such as samples in Fig. 4, the standard deviation is large and several results from different images are combined using inverse-variance weighting. For a set of data $\{ O_i\}$ and associated variance $\{ \sigma _{Oi}^2\}$, the the inverse-variance weighted average is:

$$\hat O = \mathop {\sum }\limits_i \frac{{O_i}}{{\sigma _{Oi}^2}}/\mathop {\sum }\limits_i \frac{1}{{\sigma _{Oi}^2}}$$

$$\hat \sigma _O^2 = 1/\mathop {\sum }\limits_i \frac{1}{{\sigma _{Oi}^2}}$$

Finally, a related but distinct question is to know how confident we are that an image, where an excess of variance in the angle distribution is observed, is indeed not randomly distributed. To test this null hypothesis, the normal distribution cumulative distribution function is used to get a *p* value (Supplementary Figure 7). The random variable *X*_*i*_:

$$X_i = \frac{{C_i - \mu _{{\mathrm{bin}}}}}{{\sigma _{{\mathrm{bin}}}}}$$

Is used to get the variance of the sample:

$$\sigma _x^2 = \frac{1}{{N_{{\mathrm{bin}}}}}\mathop {\sum }\limits_i^{N_{{\mathrm{bin}}}} (X_i)^2$$

which gives the following *p* value for the null hypothesis:

$$p - \mathrm{value} = 1 - \frac{1}{2}\left[ {1 + {\mathrm{erf}}\left( {\frac{{\sigma _x}}{{\sqrt 2 }}} \right)} \right]$$

A *p* value of $0.01$ means that the probability that the angles are randomly distributed is smaller than $1\%$.

### Code availability

The code for evaluating ordering of the images is available from the corresponding author.

## Electronic supplementary material

Supplementary Information

## Data Availability

The authors declare that the data supporting the findings of this study are available within the paper and its supplementary information files. The code for evaluating ordering of the images is available from the corresponding author.
