# Supplementary material for: Microfluidic deposition for resolving single-molecule protein architecture and heterogeneity
Source: Nat Commun. 2018 Sep 24;9:3890. doi: 10.1038/s41467-018-06345-4 (PMC6155325; doi:10.1038/s41467-018-06345-4)
Supplement: Supplementary file 1 — Supplementary Information [file 41467_2018_6345_MOESM1_ESM.pdf]

Microfluidic deposition for resolving single molecule protein architecture and heterogeneity

Ruggeri F.S. et al.

SUPPLEMENTARY NOTE 1

Surface interactions can bias manual AFM sample preparation

The first preparation step of sample preparation for AFM measurements is the deposition onto the surface of a droplet of sample, of about few μl, to allow physio-adsorption (Supplementary Figure 1). The adsorption requires usually a time ranging from few seconds to tens of minutes, which is several order of magnitude larger than the lateral diffusion time scale of a protein with nanoscale diameter on a liquid-solid interface. Indeed, the lateral diffusion mobility constant of a protein adsorbed on a solid-liquid interface can be estimated to be in the order of 0.2 ± 0.1 $\mu m^2 s^{-1}$, which means that the protein can move freely on the surface if the deposition time is in the order of seconds or above (Supplementary figure 2).[1-4] Thus, the process of absorption leads unavoidably to artefacts of preparation, because of self-organization and/or self-assembly of the molecules on the surface (Supplementary figure 1a). These effects are more dramatic as a function of increasing sample concentration and time of deposition, which are required to attach the molecules firmly onto the surface (Supplementary figure 3-5). The self-organization and assembly of the deposited molecules occurs following the crystalline lattice or chemical structure of the substrate. As example, in Supplementary Figure 1a on the top, we show the self-organization and assembly on monomers of α-synuclein, where the proteins follow the directions of the positive sites of etching of mica, along the 001 plane (Supplementary figure 1 and Supplementary Figure 3-4).[5] Similarly, on the bottom of Supplementary figure 1a, we show the self-organization of monomeric and oligomeric forms of Aβ42 on HOPG, where the peptides self-assembly in circles following the hexagonal symmetry of carbons sites on the surface plane (002) (Supplementary figure 1a and Supplementary Figure 5).[6] The second step of sample preparation is the rinsing of the sample by means of ultra-pure water or of a buffer solution. This operation is necessary to remove the biomolecules that are not firmly attached on the surface. The last step is the drying of the sample with a flux of nitrogen. As result of rinsing and drying, especially in the case where repulsive interactions exist between the substrate and the analyte, not all the biomolecules in solution attach on the chosen substrate, resulting in artefacts in the populations of species in heterogeneous mixtures. In Supplementary Figure 1b, we show the deposition on different surfaces of a solution of α-synuclein at an early stage of the aggregation. While we can observe only oligomeric species on the negatively charge mica (top panel), the deposition on hydrophobic HOPG enables to unravel the contemporary presence in solution of prefibrillar aggregates (bottom panel). The effect of selective absorption masks the effective heterogeneity of the protein solution leading to a biased depiction of the composition and state of aggregation of the sample. Finally, the procedures of rinsing and drying all together result in artefacts of a not reproducible control of the number and concentration of molecules deposited and dried on the surface. In Supplementary figure 1c, we show the deposition of a solution of α-synuclein containing oligomeric and elongated prefibrillar aggregated species. At sub-μM concentration of deposition, both the spherical and elongated species are adsorbed and distinguishable on the surface (Supplementary figure 1c left and Supplementary figure 6). At

increasing concentration of deposition (e.g. 2 μM), the aggregates can superimpose and overcrowd, masking the morphological properties of the individual species and the heterogeneity of the aggregated species (Supplementary figure 1c, centre). At yet higher concentrations (e.g. 15 μM), the overcrowding effect on the surface is so accentuated that only a layer of apparent oligomeric species is visible (Supplementary figure 1c, right). A measurement of the volume of the prefibrillar species as a function of the concentration of deposition shows that at low concentration of deposition the volume increases monotonically as a function of the number of species deposited; however, the presence of the prefibrillar species is not anymore detectable at high concentration of deposition (histogram, Supplementary figure 1c). Thus, a weak control of the concentration of deposition can lead to overcrowding effects masking the heterogeneity of supramolecular species populations in protein solutions.

SUPPLEMENTARY NOTE 2

Theory of sessile droplets drying

Drying during time of flight

The droplets produced by the microfluidic spray device, before landing on the surface of deposition, travel in air and partially dry during the time of flight (Supplementary figure 2). Typically, the evaporation rate of a droplet traveling in air is limited by water molecule transport across its boundary layer δ that is:[7,8]

$$\delta \approx 4.91\sqrt{\frac{\nu r}{u}} \tag{1}$$

where $\nu = 14.88 \times 10^{-6}\ \mathrm{m^2\ s^{-1}}$ is the water kinematic viscosity, $u = 20\ \mathrm{m\ s^{-1}}$ is the air velocity and r is the drop radius. For drops of radius $r = 4\ \mu m$, the boundary layer is $\delta = 8.5$ μm and the Peclet number for the water molecule transport is $= \frac{\delta u}{D_{\mathrm{H_2O}}} \approx 6$ ($D_{\mathrm{H_2O}} = 2.82 * 10^{-5}\ \mathrm{m^2\ s^{-1}}$). Therefore, the droplet evaporation rate is driven by advection and is limited by the rate at which water molecules leave the droplet surface. Kinetic theory gives the average number of molecules leaving a unit surface area:

$$\frac{dN}{dt}\frac{1}{A} = -\frac{p_{vap}}{\sqrt{2\pi m_{\mathrm{H_2O}} k_B T}} \tag{2}$$

where p_{vap} is the vapour pressure, $m_{\mathrm{H_2O}}$ is the water molecule mass, k_B is the Boltzmann constant and T is the temperature. We can estimate the molecule evaporation rate considering a thin layer of surface area A and density ρ_{W}:

$$\frac{dN}{dt} = -\frac{A\rho_{\mathrm{W}}}{m_{\mathrm{H_2O}}}\frac{dr}{dt} \tag{3}$$

The previous formula leads us to droplet radius shrinking rate:

$$\frac{dr}{dt} = -\frac{p_{\text{vap}}\sqrt{m_{\text{H}_2\text{O}}}}{\rho_{\text{W}}\sqrt{2\pi k_B T}} \tag{4}$$

In our experimental conditions, we have a vapor pressure of $p_{\text{vap}} = 962$ Pa (H = 40%, T = 20° C), which gives a droplet shrinking rate of $\dot{r} = \frac{dr}{dt} \approx -1.043 * 10^{-3}$ m s^{-1}. Assuming that the microfluidic spray device produces droplets with an initial radius R_0, we can describe equation 4 as:

$$R_{\text{A}} = R_0 - 0.001043 * t \tag{5}$$

where t is the time of flight which depends on the nozzle distance from the surface. By means of a high-speed camera, we could measure an approximate velocity of our droplets in the order of $10 - 30$ m s^{-1}.

Sessile droplet drying on a surface

The droplets landing on a surface do not experience as high air velocity, such as during spraying (Supplementary Figure 2). Therefore, the evaporation rate becomes diffusion limited. Furthermore, when the droplet lands on a surface, it does not have a spherical shape anymore. The measured typical contact angle of the droplets is 15°, thus the available surface area is increased as depicted below:

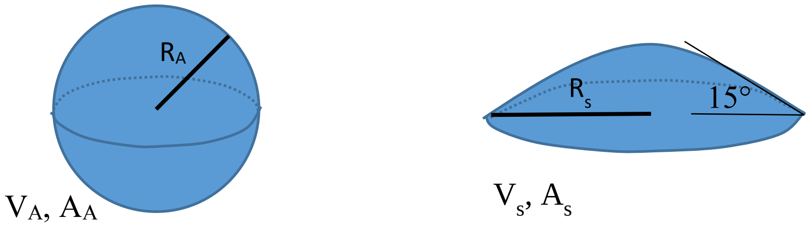

The volume $V_{\text{A}} = \frac{4}{3}\pi R_{\text{A}}^3$ and the surface area $A_{\text{A}} = 4\pi R_{\text{A}}^2$ of the droplet in air becomes $V_{\text{s}} \approx \frac{\pi}{4} R_{\text{s}}^3\theta$ and $A_{\text{s}} \approx \pi R_{\text{s}}^2$ on the surface. We can then consider that approximately $R_{\text{s}} \approx 2.7R_{\text{A}}$ and $A_{\text{s}} \approx 1.84\, A_{\text{A}}$.

Considering a spherical evaporation model, proposed by Maxwell,[9,10] the change in mass of a droplet is:

$$R_0^2 - R(t)^2 = \frac{2DM}{\rho RT}(P_{\text{s}} - P_\infty)t \tag{6}$$

Where R_0 is the initial radius, M is the molecular weight (g·mol^{-1}), T the temperature (K), $\mathfrak{R}$ the gas constant ($\mathfrak{R}$=8.31 J·K^{-1}·mol^{-1}), D is the vapor diffusion constant in air, ρ is the density of the liquid, P_S the partial pressure at the surface, and P_∞ the infinite partial pressure. Assuming the droplet evaporation on the surface is still diffusion limited, we can approximate the droplet radius as a function of time as the d^2 law for evaporation[4]:

$$R(t)^2 = R_s^2 - \beta t \qquad (7)$$

In the case of fluorescein, for droplets sprayed from a distance of 2 cm from the surface, the median volume on the surface is $V_s \approx 100$ fL ($R_s = 7.8$ µm, $R_A = 2.9$ µm), the interquartile range of the droplet volume distribution is $V_{range} \approx 40 - 210\ fL$ ($R_s = 5.7 - 10$ µm), and the evaporation time is $t_s = 5$ ms, as measured by high-speed imaging experiment. Thus, we estimated $\beta = 1.20 * 10^{-8}\ \mathrm{m^2 s^{-1}}$.

We can now estimate what is the average initial radius R_0 and size of the droplets generated by the spray. Using equation 5:

$$R_0 = R_A(2.9\ \mu m) + \dot{r}(0.001034) * t_{2cm}(1\mathrm{ms}) = 3.9\ \mu\mathrm{m}$$

where the time between the droplet generation and landing on the surface is around $t_{2cm} = \frac{2\ \mathrm{cm}}{20\ \mathrm{m\ s^{-1}}} = 1$ ms. The initial median volume of the droplets at the exit of the microfluidic spray device is $V_0 = 250$ fL, with the interquartile range of $130 - 450$ fL ($R_A = 3.2 - 4.8$ µm).

In our experiments the analyte solution is sprayed on the surface at a distance of $h = 4$ cm away from the surface (see figure 1), therefore, the spherical droplet radius just before landing is $R_{A\ @\ 4cm} = 1.9$ µm which on the surface is of radius $R_{S\ @\ 4cm} = 4.9$ µm and takes about $t_{S\ @\ 4cm} = 2$ ms to evaporate. The interquartile range of the surface droplet radius, volume and the drying time is $R_{range} = 2.9 - 7.2$ µm, $V_{range} = 5 - 80$ fL and $t_{range} = 0.7 - 4.3$ ms.

Effect of temperature and humidity on the time of drying

Droplet evaporation rate depends weakly on temperature and the relative humidity. Our measurements are performed in a laboratory with constant temperature and relative humidity control by a typical air conditioning system, which maintain $T = 20 \pm 2°C$ and $\Delta H = 40 \pm 10\%$ that results in a water vapour pressure of $p_{vap} = 960 \pm 150$ Pa. The latter variations in the vapour pressure, combined with an error in the nozzle positioning of ± 1 mm, the surface lead to the average droplet size landing on the surface $R_S = 4.9 \pm 0.9\ \mu m$ and a related time of drying time is $t_s = 2 \pm 0.8$ ms. The variation of the time of drying upon changes in temperature and humidity is of approximately 40%, as represented by the error bars in R_S and t_S. This value is smaller than the interquartile range variation of the time of drying of the droplets, due to their different size. Therefore, temperature and humidity do not play an important role during the sample deposition on surface.

SUPPLEMENTARY NOTE 3

Molecular diffusion on a surface

We can consider the pure translational diffusion in 2-D as the superior limit of lateral diffusion for a single protein in liquid on a solid surface. We can consider a typical molecular diffusion constant on surface of $D_{\text{surf}} = 0.2 \pm 0.1\ \mu\text{m}^2\ \text{s}^{-1}$. After the droplet deposition, molecules, attached to a surface, can diffuse a distance:

$$\Delta r = 2\sqrt{\text{D}_{\text{surf}} \cdot t_{\text{S@4cm}}}$$

Where D_{surf} is the diffusion constant and t_s the time of drying. This gives a typical molecular diffusion distance in the range $\Delta r \approx 40 \pm 20$ nm (Supplementary figure 2), considering the measured droplet size distribution (Figure 2). The diffusion length is calculated considering that the protein are in contact with the surface for the full time of drying, thus the 2D diffusion length can be considered as extreme and unfavorable superior limit cases of diffusion. Indeed, we are not considering that once a droplet lands on the surface, first proteins need to diffuse towards the interface and only later the 2-D diffusion can occur. The 3-D diffusion of protein in water solution has a characteristic diffusion constant in the order of 1-10 $\mu\text{m}^2\ \text{s}^{-1}$ for typical scale of the droplets in the order of few micrometers, thus this phenomena occurs at a similar time scale of drying for sub-picoliter droplets and reduces the effective time available during the drying for the 2-D diffusion of proteins at the solid-liquid interface.[3,11]

SUPPLEMENTARY NOTE 4

Average distance of molecules within a deposited droplet

First, we estimate the total number of molecules in one droplet of volume V_0 and R_0 radius in air:

$$N = cV_0N_{\text{A}} = c\frac{4}{3}\pi R_0^3 N_{\text{A}}$$

Where c is the concentration and N_a is the Avogadro number. Then, we can estimate the average molecular spacing d on a surface by fitting N disks of radius d/2 in an area of the droplet on the surface with radius R_S:

$$d = \frac{2R_{\text{S}}}{\sqrt{N}} = \frac{2R_{\text{S}}}{\sqrt{c\frac{4}{3}\pi R_0^3 N_{\text{A}}}}$$

For a concentration of $c_{0.2} = 0.2\ \mu\text{M}$ the spacing is $d_{0.2} = 57 \pm 12$ nm whereas for a concentration of $c_2 = 2\mu M$ the spacing is $d_2 = 18 \pm 4$ nm.

SUPPLEMENTARY NOTE 5

Surface coverage of the droplets upon spraying

The spray is placed roughly $H = 4$ cm above the surface and the spray angle is measured to be 16.5°. The area covered by the droplet jet is $A_T = 4.4\ \text{cm}^2$. We can estimate, the number of droplets generated in time t at a flow rate Q by considering the average droplet size $R_0 = 3.9$ µm of volume $V_0 = 250$ fL:

$$N_{\text{drops}} = \frac{V_{\text{spray}}}{V_0} = \frac{Q\ t}{V_0}$$

The droplets evaporate during the time of flight and upon landing form circles of radius $R_S = 4.9$ µm. Total surface coverage during total spray time $t = 5$ s and a flow rate $Q = 100$ µL h^{-1} is:

$$\eta = \frac{N_{\text{drops}} \pi R_S^2}{A_T} = 9.7\%$$

We can conclude that for a time of spraying below 30 seconds the droplets are unlikely to overlap on the deposition surface. Thus, the molecule observed on the surface derive from the content of a single droplet.

SUPPLEMENTARY NOTE 6

Quartz microbalance measurement of droplets drying

To prove that droplets indeed dry and do not have any residual water left, we have sprayed deionised water and 10 mM sodium phosphate solutions at a 200 µL h^{-1} flow rate on a Quartz Crystal Microbalance (QCM200 - 5 MHz quartz crystal microbalance from Stanford Research Systems). We have observed no deposition for the water solution whereas the sodium phosphate solution showed a linear frequency decrease indicating continuous mass deposition, as showed in Supplementary figure 11.[12] This means that indeed the droplet evaporate on the surface accumulating the solute on the surface.

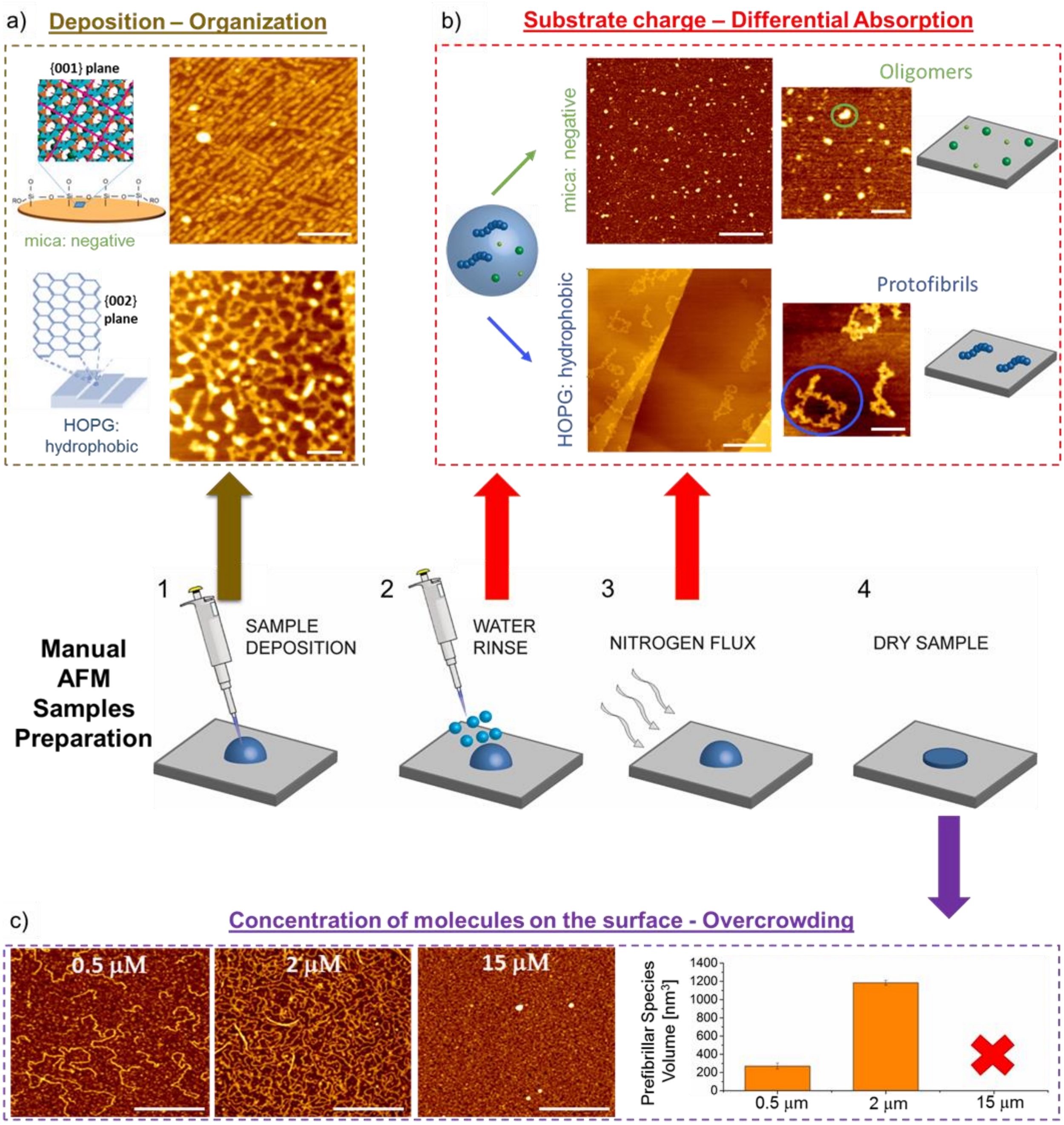

Supplementary Figure 1. Artefacts of manual preparation of AFM samples. *At the centre, a schematic representation of the sample preparation procedure is presented: 1) sample deposition, 2) water rinsing, 3) drying. a) Long deposition times cause self-organization of monomeric α-synuclein on mica (top) and on HOPG (bottom) (scale bar 100 nm). The self-organized structures follow the main crystallographic axes of the two substrate*[13]*. b) Because of surface selectivity, diverse amyloidogenic species of α-synuclein are visible on negative mica (oligomers, top) and hydrophobic HOPG (protofibrillar species, bottom (scale bars 200 nm for the images on the left, 60 nm for the images on the right). (c) Overcrowding of α-synuclein aggregates on mica surface. The same solution is deposited at different concentrations. A low concentration of deposition (0.2 µM) allows visualizing fibrillar species, these species superimpose and overcrowd at increasing concentrations (e.g. 2 µM) and are not visible at higher concentrations (e.g. 15 µM) because a complete layer of aggregates is covering the surface. The effect of overcrowding is quantified by measuring the total volume of fibrillar species on the surface, at 15 µm the elongated species are not measurable because of the formation of a layer (values are mean ± s.d; scale bars 200 nm).*

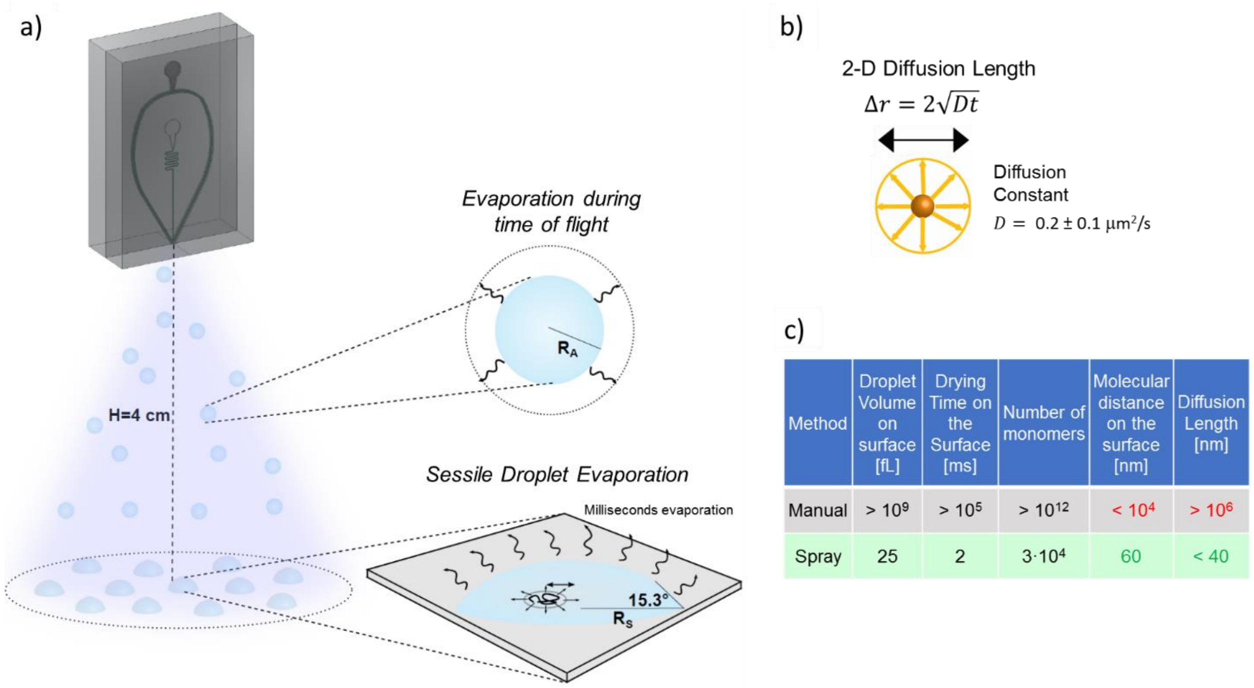

Method	Droplet Volume on surface [fL]	Drying Time on the Surface [ms]	Number of monomers	Molecular distance on the surface [nm]	Diffusion Length [nm]
Manual	$> 10^9$	$> 10^5$	$> 10^{12}$	$< 10^4$	$> 10^6$
Spray	25	2	$3 \cdot 10^4$	60	< 40

Supplementary Figure 2. Microfluidic spray and model of pure lateral translational diffusion of a protein on a solid surface. *a) The microfluidic device transfers a biological solution on a surface in the form of microdroplets with volume ranging between 5-80 fL (first-third quartile range). The droplets first evaporate during the time of flight and then evaporate as sessile droplet on a substrate. The particle is depicted on a liquid-solid interface as a relaxed polypeptide chain, which represent a monomeric intrinsically disordered protein on a surface. b) Solution of pure translational diffusion in 2-D, considered as the superior limit of diffusion for a single protein in liquid on a solid surface, where Δr is the distance covered, D the diffusion constant that is typically within the range 0.2 ± 0.1 $\mu m^2\ s^{-1}$ and t is the time. c) We report the comparison of the key parameters during drying in the case of manual and spray deposition of monomeric α-synuclein at 0.2 μM. We report the median volume, evaporation time of a droplet[14], the number of molecules, their approximated average intermolecular spacing and the relative superior limit of diffusion length of the protein on the surface during the time of drying. Remarkably, in the case of manual deposition the molecular distance is much smaller than diffusion length, allowing self-organization and self-aggregation on the surface. While in the case of microfluidic spray, deposition the molecular distance is bigger than the diffusion length. The diffusion length is calculated considering that the protein are in contact with the surface for the full time of drying, thus the values in the table can be considered as extreme and unfavorable superior limit cases of diffusion. Indeed, we are not considering that once a droplet lands on the surface, first proteins need to diffuse towards the interface and only later the 2-D diffusion can occur. The 3-D diffusion of protein in water solution has a characteristic diffusion constant in the order of 1-10 $\mu m^2\ s^{-1}$ for typical scale of the droplets in the order of few micrometers, thus this phenomena occurs at a similar time scale of drying for sub-picoliter droplets and reduces the effective time available during the drying for the 2-D diffusion of proteins at the solid-liquid interface.[3,11]*

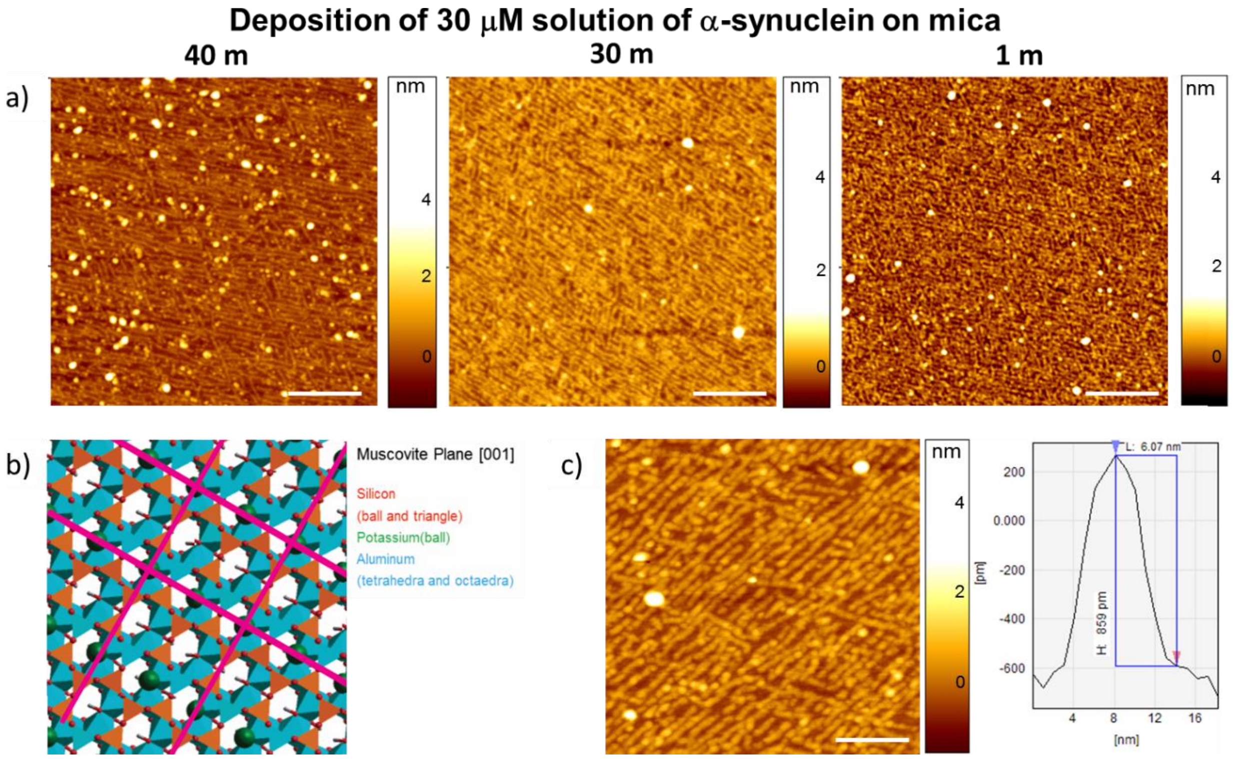

Supplementary Figure 3. Time dependence of the self-organization of α-synuclein monomers on mica. *(a) Self-organization as a function of the time of deposition (40, 30 and 1 min at a fixed concentration of 30 μM (scale bars 200 nm). (b) Schematic illustration of mica crystal order; the potassium sites (green spheres) indicate the ordering of the negative sites of mica on the surface (scale bars 200 nm). c) High-resolution detail of the self-assembly of monomers following the direction of negative sites on mica (scale bar 100 nm).*

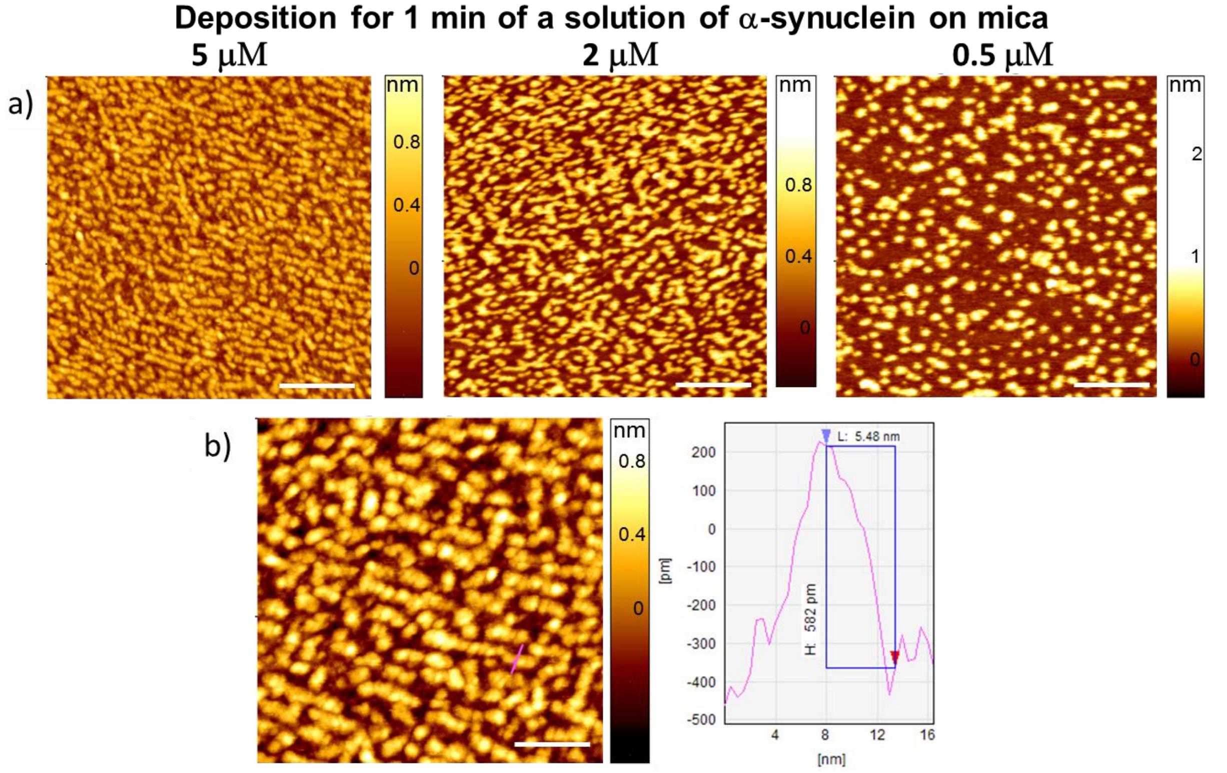

Supplementary Figure 4. Concentration dependence of the self-organization of α-synuclein monomers on mica. *(a) Self-organization as a function of the concentration of deposition (5, 2 and 0.5 μM with a deposition time of 1 min) (scale bar 100 nm). b) High-resolution detail of the monomers self-assembly following the direction of negative sites on mica (5 μM) (scale bar 50 nm).*

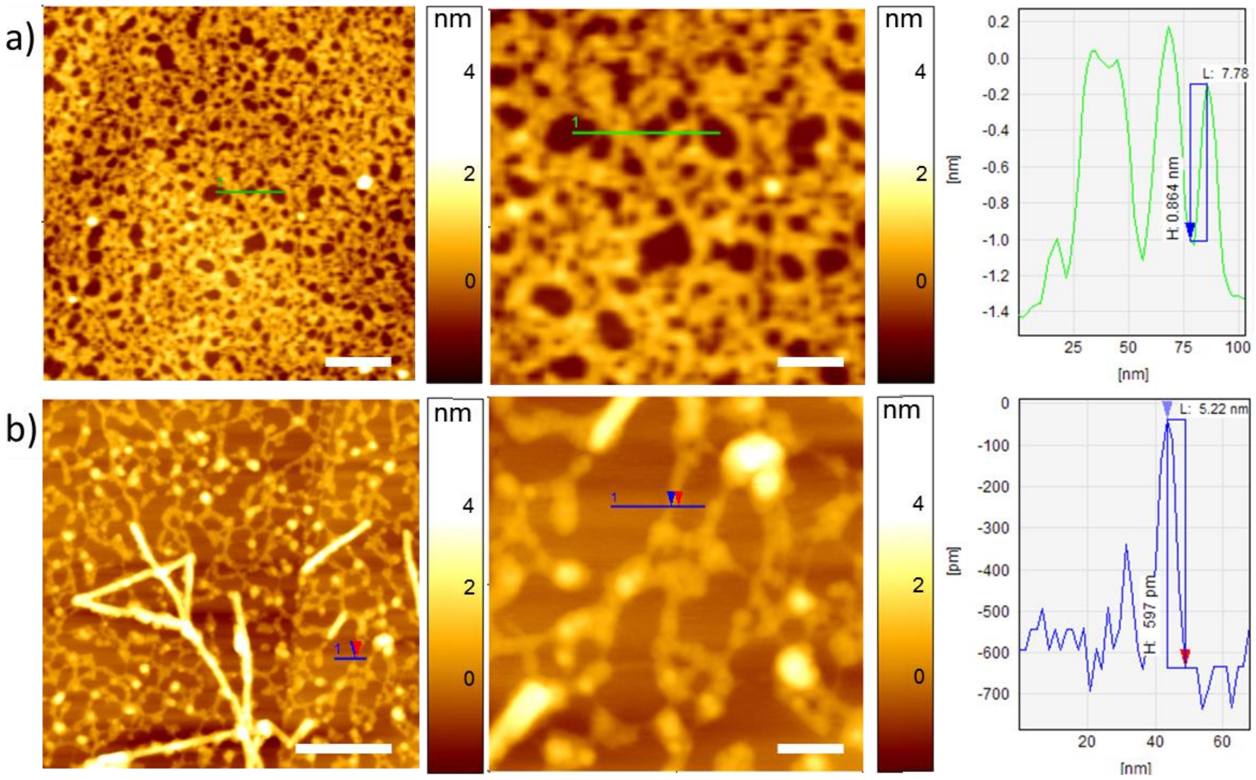

Supplementary Figure 5*. *Self-organization of α-synuclein on HOPG. *(a) Solution of monomers deposited at a concentration of 2 μM for 1 min and (b) a mixture of monomers, oligomers and fibrils on HOPG deposited at a concentration of 10 μM for 10 min.*

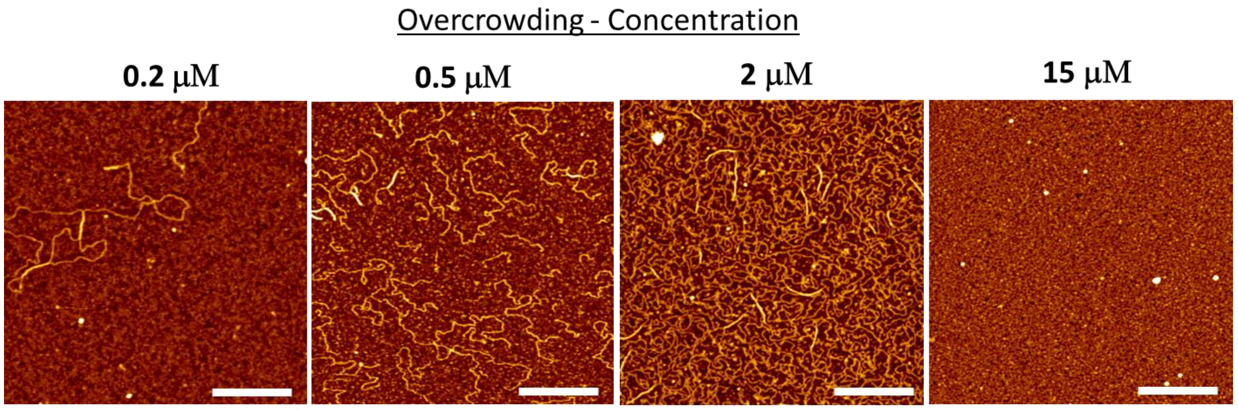

Supplementary Figure 6. Effects of overcrowding as a function of concentration. *Early protofibrillar aggregates of α-synuclein crowd the surface. High concentration of proteins creates a complete layer on the surface, which does not enables the elongated species to be visualized. All the samples were deposited on the surface for 1 min. (scale bars 200 nm)*

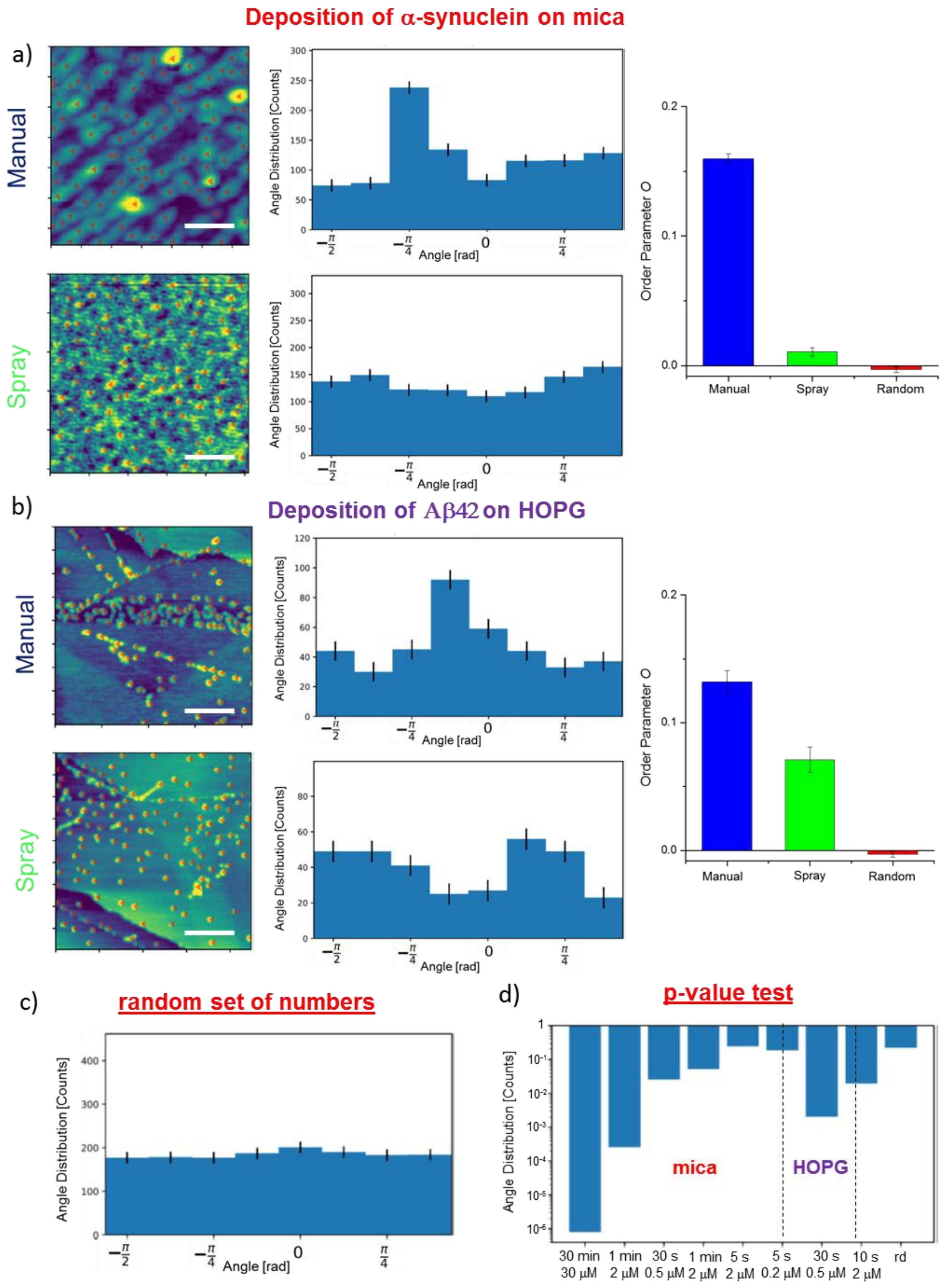

Supplementary Figure 7. Example of evaluation of ordering in AFM maps of deposited proteins. *Monomers of a) α-synuclein on MICA (scale bars 50 nm), b) Aβ42 on HOPG (scale bars 100 nm). On the left of the images are showed zoomed areas of Figure 3, where proteins are localized. At the center are shown the histograms of the close neighbor's angles. On the right, the degree of ordering plots are calculated as the normalized sum of the areas of the maxima related to the ordering along a direction. c) As control, the histogram of excess angle for a random set, representing a randomly distributed sample on a surface, is shown and compared to the data relative to the protein (errors are s.d.). d) p-value test evaluating the null hypothesis that the molecules on the surface are not randomly organised as calculated from the formula defined in the methods section of the manuscript:* $p - value = 1 - 1/2\ [1 + erf(\sigma\_x/\sqrt{2})]$.

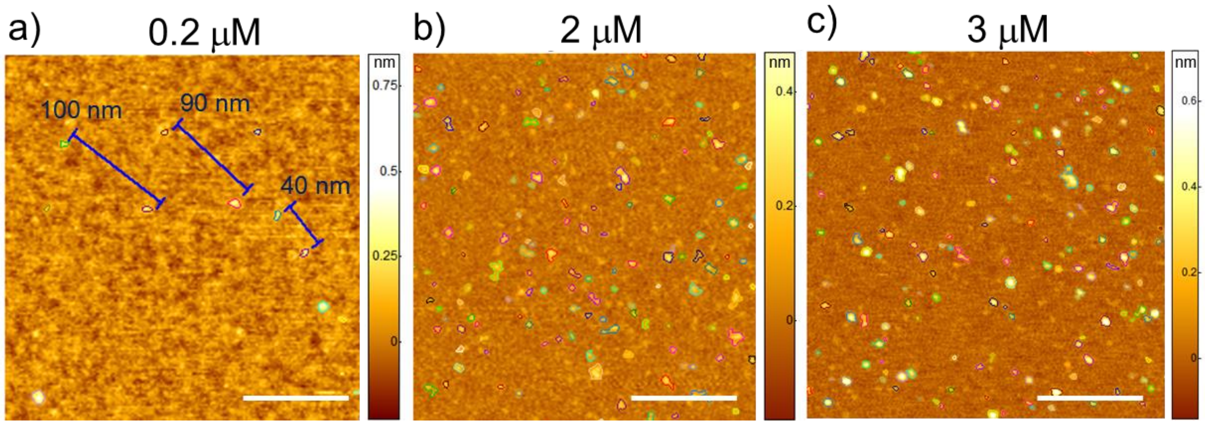

Supplementary Figure 8. Microfluidic spray device preserves molecular architecture. *Deposition of α–synuclein monomers at as a function of increasing concentration of a) 0.2, b) 2 and c) 3 μm. c) The colored circles represent the molecules on the surface and the blue line examples of distance between two molecules. (Scale bars 200 nm)*

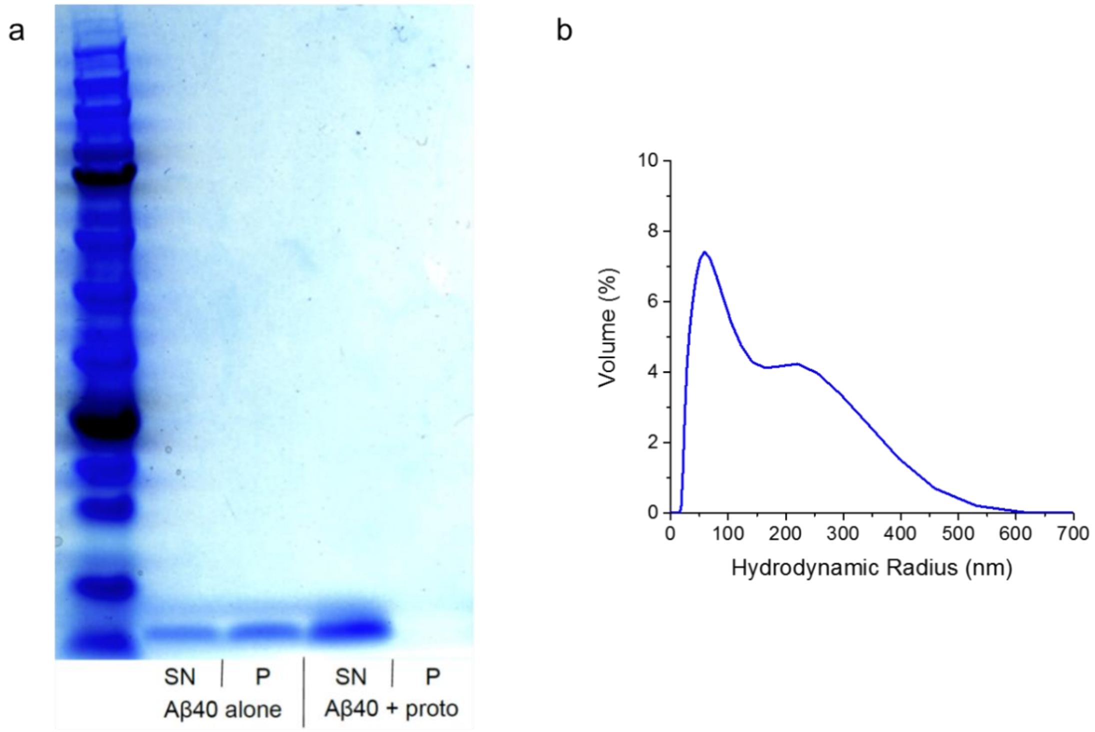

Supplementary Figure 9. SDS-PAGE and DLS analysis of ab40 aggregation. *a) Supernatant (SN) and pellet (P) fractions obtained after centrifugation of samples of Aβ40 that aggregated in the absence and presence of protoporphyrin. b) Dynamic light scattering (DLS) measurement of the Aβ40 plus protoporphyrin sample shows the presence of heterogeneous species with hydrodynamic radius between 50-500 nm.*

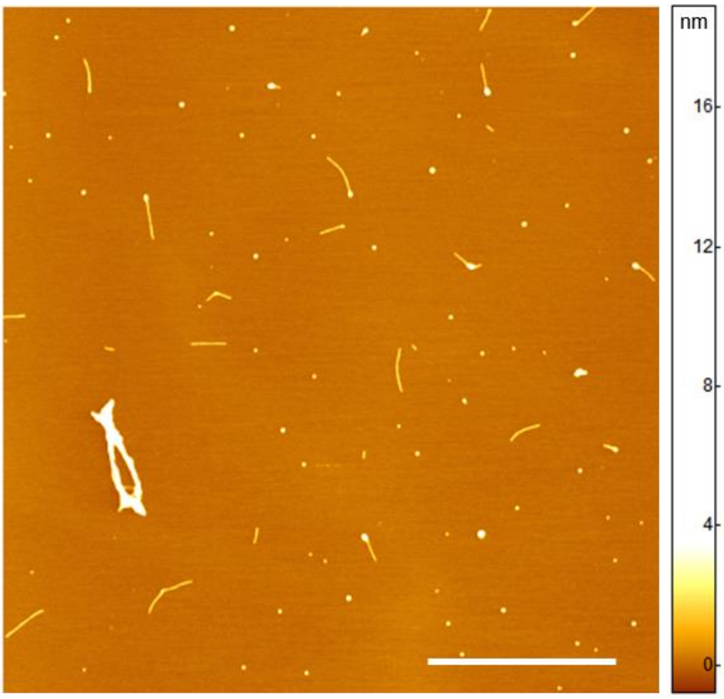

Supplementary Figure 10. AFM image of manual deposition of Aβ40 fibrils on mica. *(Scale bar 2 μm)*

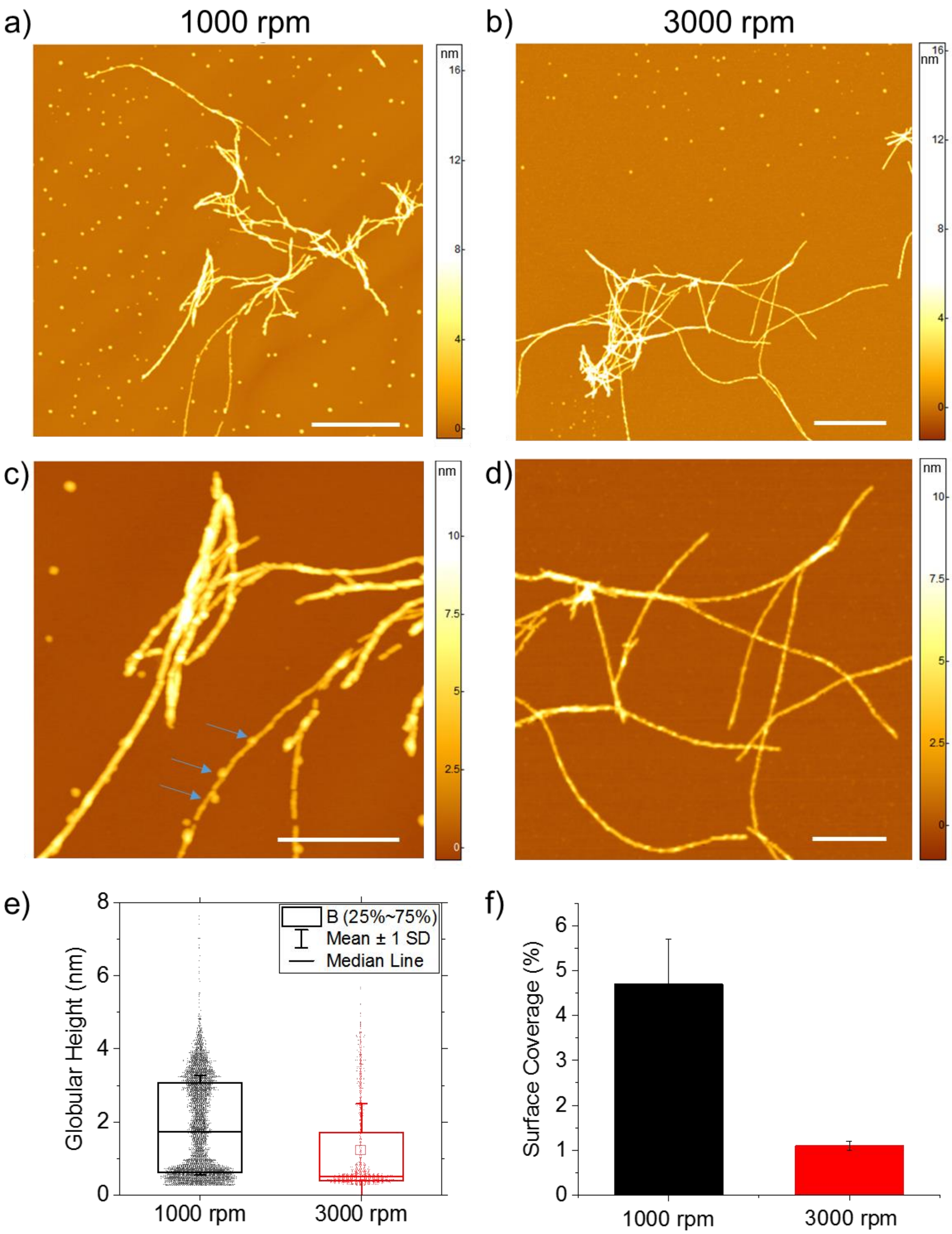

Supplementary Figure 11. Artificial formation of globular species upon spin coating deposition of Aβ42 fibrils. *a,b) AFM maps of fibrils deposited at 1000 and 3000 rpm (scale bars 1 μm). c,d) Detail of the maps showing the coverage of the substrates and of the fibrils by artificial globular species (scale bars 400 nm). The analysis of the globular species shows that they change e) diameter (n=7015 for 1000 rpm and n=1349 for 3000 rpm, the horizontal line represents the median, the box the 1st-3rd interquartile and the error is the s.d.) and f) abundancy on the surface as a function of the spin speed (values are mean ± s.d.).*

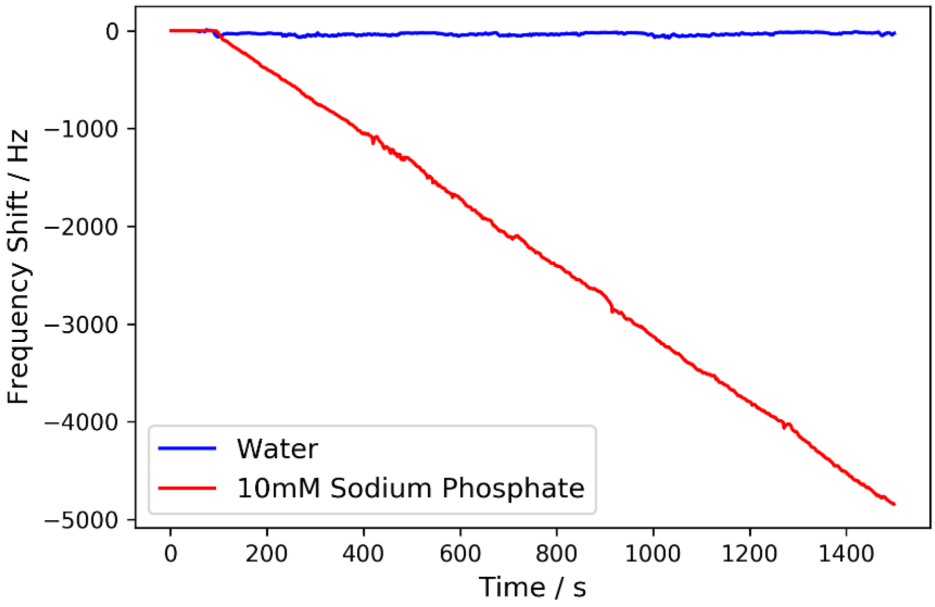

Supplementary Figure 12. Quartz microbalance measurements of droplets drying. *Continuous deposition of water and 10 mM sodium phosphate solution at* 200 $\mu l\ h^{-1}$ *indicate that droplets evaporate on surface leaving only the dry solute on the surface.*

SUPPLEMENTARY REFERENCES

1 Tilton, R. D., Gast, A. P. & Robertson, C. R. Surface diffusion of interacting proteins. Effect of concentration on the lateral mobility of adsorbed bovine serum albumin. *Biophysical Journal* **58**, 1321-1326, (1990).

2 Ravichandran, S. & Talbot, J. Mobility of Adsorbed Proteins: A Brownian Dynamics Study. *Biophysical Journal* **78**, 110-120, (2000).

3 Chan, V., Graves, D. J., Fortina, P. & McKenzie, S. E. Adsorption and Surface Diffusion of DNA Oligonucleotides at Liquid/Solid Interfaces. *Langmuir* **13**, 320-329, (1997).

4 Zhao, J. & Granick, S. Polymer Lateral Diffusion at the Solid−Liquid Interface. *Journal of the American Chemical Society* **126**, 6242-6243, (2004).

5 Ostendorf, F. *et al.* How flat is an air-cleaved mica surface? *Nanotechnology* **19**, 305705, (2008).

6 Patel, A. N. *et al.* A New View of Electrochemistry at Highly Oriented Pyrolytic Graphite. *Journal of the American Chemical Society* **134**, 20117-20130, (2012).

7 Amstad, E. *et al.* Production of amorphous nanoparticles by supersonic spray-drying with a microfluidic nebulator. *Science* **349**, 956-960, (2015).

8 Amstad, E., Spaepen, F., Brenner, M. P. & Weitz, D. A. The microfluidic nebulator: production of sub-micrometer sized airborne drops. *Lab Chip* **17**, 1475-1480, (2017).

9 Sadek, C. *et al.* Drying of a single droplet to investigate process-structure-function relationships: a review. *Dairy Science & Technology* **95**, 771-794, (2015).

10 Law, C. K. Recent advances in droplet vaporization and combustion. *Progress in Energy and Combustion Science* **8**, 171-201, (1982).

11 Latour, R. A. The langmuir isotherm: A commonly applied but misleading approach for the analysis of protein adsorption behavior. *Journal of Biomedical Materials Research Part A* **103**, 949-958, (2015).

12 O'Sullivan, C. K. & Guilbault, G. G. Commercial quartz crystal microbalances – theory and applications. *Biosensors and Bioelectronics* **14**, 663-670, (1999).

13 Losic, D., Martin, L. L., Aguilar, M. I. & Small, D. H. Beta-amyloid fibril formation is promoted by step edges of highly oriented pyrolytic graphite. *Biopolymers* **84**, 519-526, (2006).

14 Larson, R. G. Transport and deposition patterns in drying sessile droplets. *AIChE Journal* **60**, 1538-1571, (2014).
